# Application of Polymers for Chemical Enhanced Oil Recovery: A Review

**DOI:** 10.3390/polym14071433

**Published:** 2022-03-31

**Authors:** Afeez Gbadamosi, Shirish Patil, Muhammad Shahzad Kamal, Ahmad A. Adewunmi, Adeyinka S. Yusuff, Augustine Agi, Jeffrey Oseh

**Affiliations:** 1 Department of Petroleum Engineering, College of Petroleum and Geosciences, King Fahd University of Petroleum and Minerals, Dhahran 31261, Saudi Arabia; a.gbadamosi@kfupm.edu.sa; 2 Centre for Integrative Petroleum Research, College of Petroleum and Geosciences, King Fahd University of Petroleum and Minerals, Dhahran 31261, Saudi Arabia; shahzadmalik@kfupm.edu.sa (M.S.K.); ahmadade@kfupm.edu.sa (A.A.A.); 3 Department of Chemical and Petroleum Engineering, Afe Babalola University, Ado-Ekiti PMB 5454, Nigeria; yusuffas@abuad.edu.ng; 4 Department of Petroleum Engineering, School of Chemical and Energy Engineering, Faculty of Engineering, Universiti Teknologi Malaysia, Johor Bahru 81310, Malaysia; aaagi2@live.utm.my; 5 Department of Petroleum Engineering, School of Engineering and Engineering Technology, Federal University of Technology, Owerri PMB 1526, Nigeria; jeffrey.oseh@futo.edu.ng

**Keywords:** polymer, rheology, polyacrylamide, biopolymer, enhanced oil recovery, hydrophobically associating polyacrylamide

## Abstract

Polymers play a significant role in enhanced oil recovery (EOR) due to their viscoelastic properties and macromolecular structure. Herein, the mechanisms of the application of polymeric materials for enhanced oil recovery are elucidated. Subsequently, the polymer types used for EOR, namely synthetic polymers and natural polymers (biopolymers), and their properties are discussed. Moreover, the numerous applications for EOR such as polymer flooding, polymer foam flooding, alkali–polymer flooding, surfactant–polymer flooding, alkali–surfactant–polymer flooding, and polymeric nanofluid flooding are appraised and evaluated. Most of the polymers exhibit pseudoplastic behavior in the presence of shear forces. The biopolymers exhibit better salt tolerance and thermal stability but are susceptible to plugging and biodegradation. As for associative synthetic polyacrylamide, several complexities are involved in unlocking its full potential. Hence, hydrolyzed polyacrylamide remains the most coveted polymer for field application of polymer floods. Finally, alkali–surfactant–polymer flooding shows good efficiency at pilot and field scales, while a recently devised polymeric nanofluid shows good potential for field application of polymer flooding for EOR.

## 1. Introduction

After the application of primary and secondary recovery, the literature suggests huge volumes of oil remain in a reservoir [1]. The remaining oil-in-place is the target of enhanced oil recovery (EOR). Hence, EOR methods are used for recovering bypassed and residual oil in the reservoir [2,3]. The devised EOR methods are majorly classified into thermal and nonthermal EOR. Thermal EOR is unsuitable for reservoirs with huge depths, thin pay zones, or underlying aquifers. This is because of high heat loss to overburden and underburden layers [4]. More importantly, the application of thermal EOR is limited due to huge concerns associated with large emissions of greenhouse gases which can lead to global warming and climate change [5]. Hence, nonthermal EOR has received prodigious attention for the recovery of conventional and heavy oil.

Chemical EOR, a nonthermal EOR method, has been proffered to improve oil recovery due to its ease of application and high efficiency. Several chemicals such as alkalis, surfactants, nanoparticles, and polymers have been utilized for EOR [6,7,8,9]. The chemicals tune the fluid–fluid and/or rock–fluid properties of the reservoir to aid oil recovery. Depending on the type of chemical utilized, the fluid–fluid and/or rock–fluid interaction causes a higher pore-scale displacement efficiency or enhances the sweep efficiency in the reservoir. Of the numerous chemical EOR methods, polymers have distinct properties and high efficiency. In fact, numerous field applications of polymer flooding have been reported in Daqing oil field in China, Pelican Lake in Canada, West Cat Canyon field in the USA, and others [10,11].

Polymers are viscoelastic in nature with pseudoplastic and shear thickening behavior when subjected to shear stress in porous media. The application of polymers improves the viscosity of the injectant, thereby causing a favorable mobility ratio in the reservoir [12]. Hence, unswept and bypassed oil in the reservoir is recovered by minimizing and/or eradicating viscous fingering, and a higher oil recovery efficiency is achieved. Besides, due to the viscoelastic structure of the macromolecular structure of polymers, they can recover oil films in constricted places in the reservoir via pulling and stripping mechanisms [13]. Additionally, polymers enhance oil recovery via the mechanism of disproportionate permeability reduction by swelling and reducing the permeability of water [14].

Furthermore, the use of polymers for EOR means a significant reduction in the amount of water required to be injected into reservoirs. Moreover, the presence of polymers also reduces the water cut in production wells. The reduction in water requirement is a significant contribution in onshore wells and deserts with minimal water availability, while the reduction in the water cut of production wells is essential in offshore wells where produced water must be treated to certain specifications prior to reuse or disposal in water bodies. Numerous polymers have been appraised for EOR. The polymers used for EOR are broadly categorized into natural polymers (biopolymers) and synthetic polymers [15,16].

Biopolymers are usually derived from natural plant products; thus, they are termed eco-friendly. They consist of monomeric sugars joined together by O-glycosidic linkages, hence forming a larger structure [17]. The characteristic of a biopolymer is determined by the properties of the monomers, linkages, and chemical modifications. Moreover, biopolymers exhibit a super thickening effect and are of low cost [18]. The raw materials are available in large quantities, and the processes of extraction and processing of the polymers by large-scale fermentation are relatively inexpensive. The product is a flexible macromolecular structure that gives room for modification and versatile use of the polymeric material for the oil recovery process. Xanthan gum, guar gum, cellulose, schizophyllan, lignin, and mushroom polysaccharide are typical examples of biopolymers evaluated for EOR. Biopolymers are mostly stable in high-salinity and high-temperature conditions. Nonetheless, the major limitations of biopolymers are oxidation, bacterial degradation, and risk associated with plugging [16].

On the other hand, acrylamide-based polymers are synthetic polymers used for EOR. They demonstrate excellent rheology and viscoelastic properties. Acrylamide-based polymers possess carboxylate and amide groups on the polymer backbone. Partially hydrolyzed polyacrylamide (HPAM), a synthetic polymer, is widely regarded as the most used polymer for field application [19]. Other synthetic polymers include polyacrylamide (PAM), and hydrophobically associating polyacrylamide (HAPAM). Nonetheless, synthetic polymers are susceptible to high-salinity and high-hardness brine, low pH, high shear rate, and high-temperature condition [20].

Recent applications of polymers for EOR are categorized into two main types. Firstly, polymers are used as a standalone treatment to achieve optimum oil recovery, also referred to as polymer flooding. Beyond that, due to the structure–property relationship of polymeric materials, they are also used for stabilizing foams, alkalis, surfactants, and more recently nanoparticles. These are termed polymer foam flooding, alkali–polymer flooding, surfactant–polymer flooding, and polymeric nanofluid flooding, respectively. Herein, recent developments in the application of polymers for EOR are discussed in detail. Firstly, the mechanisms of polymer flooding application for improving oil recovery are enumerated. Subsequently, the properties of the various biopolymers and synthetic polymers used for EOR are highlighted. Then, the influence of critical parameters on the polymer is discussed. Finally, the various polymer EOR methods are evaluated.

## 2. Mechanisms of Polymer Applications for EOR

### 2.1. Mobility Ratio

Mobility ratio is referred to as the ratio of the displacing fluid (i.e., water) mobility to the displaced fluid (oil) mobility. In a typical waterflooding scenario, the mobility ratio (M) is expressed as follows:

(1) M=λwλo=krwμwkroμo=krwμokroμw    

where *λ_w_* is the mobility of water, *λ_o_* is the mobility of oil, *k_rw_* is the relative permeability of water, *k_ro_* is the relative permeability of oil, *μ_w_* is the viscosity of water, and *μ_o_* is the viscosity of oil. 

**Figure 1:**
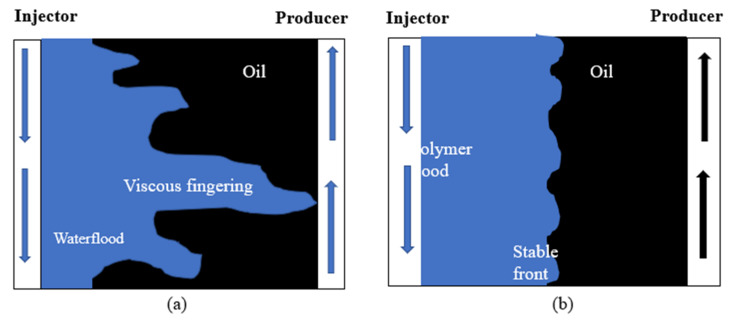
(**a**) Waterflooding process (M > 1.0); (**b**) polymer flooding process (M < 1.0) [20].

**Figure 2:**
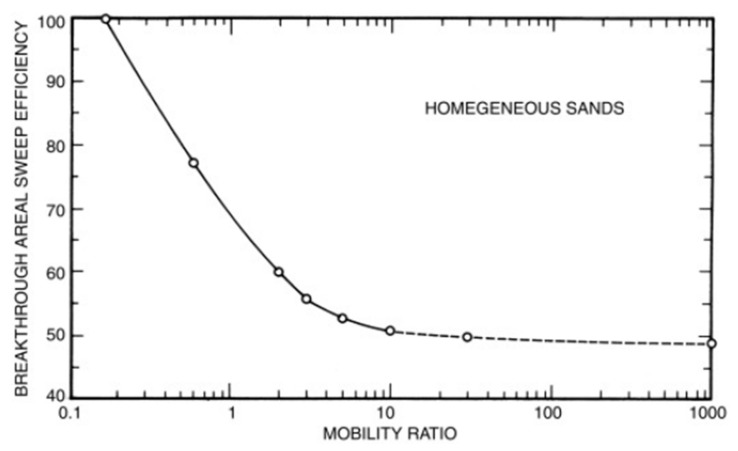
Effect of mobility ratio on sweep efficiency [17].

More importantly, M is an indication of the stability of the displacement process during oil recovery. During waterflooding, injected water tends to follow the path of least resistance, thereby creating a viscous fingering phenomenon as depicted in Figure 1a. This implies there is a large viscosity difference between displacing fluid (water) and the displaced fluid (oil) (i.e., M > 1.0). Hence, the presence of the nonuniform displacement front causes a huge volume of oil to be bypassed in the reservoir [20]. It is usually desirable to lower the mobility of water with respect to oil in the reservoir. When M < 1.0 (see Figure 1b), this means a stable displacement front is formed which minimizes and/or eradicates viscous fingering. Resultantly, enough of the injectant mobilizes and pushes the oil toward the production well. The addition of water-soluble polymers into injected water flood thickens and improves the viscosity of the injectant. Consequently, the fractional flow of water decreases (i.e., the mobility of the injectant is lowered), thereby causing a high volumetric sweep efficiency as depicted in Figure 2 [21,22].

### 2.2. Disproportionate Permeability Reduction

Disproportionate permeability reduction (DPR) is another mechanism by which polymer flood improves oil recovery. Generally, most oil reservoirs have a heterogeneous structure characterized by varying permeabilities in several layers. During waterflooding, water channels through the high-permeability regions of the reservoir, leading to high water-cut. Resultantly, huge amounts of oil and gas are trapped in low-permeability regions, and lower oil recovery is achieved. This situation is remedied with the introduction of a polymer flood. In a water-wet reservoir, the injection of polymer solution causes the formation of a thin layer on the reservoir rock due to adsorption. The adsorbed polymer film swells when it comes in contact with water, thus resisting its flow while allowing the flow of oil [23]. Additionally, the tails, loops, and protruding ends of the flowing polymer become entangled with the adsorbed polymer, thus reducing the area available for the water to flow [24]. The resistance built to water flow diverts the subsequently injected water to unswept regions of the reservoir, thereby improving oil recovery.

### 2.3. Viscoelasticity

Generally, polymers used for EOR are viscoelastic in nature. Injected polymer solutions are subjected to varying shear rates during their propagation in the reservoir. Due to their viscoelastic nature, the macromolecules of the polymer expand and contract by stretching and recoiling when flowing in porous media. This phenomenon displayed by polymers improves sweep and displacement efficiency [25]. Pulling, stripping, oil thread mobilization, and shear thickening effect have been identified as the mechanisms responsible for the viscoelastic effect of polymers on oil mobilization [13,26]. Wang et al. [27] extensively studied the flow behavior of a viscoelastic polymer solution on oil displacement efficiency. They showed that the residual oil after waterflooding is decreased by the pulling effect. The larger the viscoelastic property of the polymer solution, the higher the efficiency of the polymer solution to sweep out oil in dead ends. Moreover, a new channel for oil flow, referred to as “oil thread”, was observed.

## 3. Polymers Utilized for EOR

Several types of polymers have been appraised and evaluated for EOR both at the laboratory scale and in field application. Broadly, polymers used for EOR are classified into natural and synthetic polymers.

### 3.1. Natural Polymers (Biopolymers)

Natural polymers, also commonly referred to as biopolymers, are polymers synthesized from natural plants or bioproducts. Examples are xanthan gum, guar gum, welan gum, scleroglucan, cellulose, schizophyllan, lignin, and mushroom polysaccharide. The gums are a group of polysaccharides that yield viscous solutions when dissolved in water at low concentrations.

#### 3.1.1. Xanthan Gum

**Figure 3:**
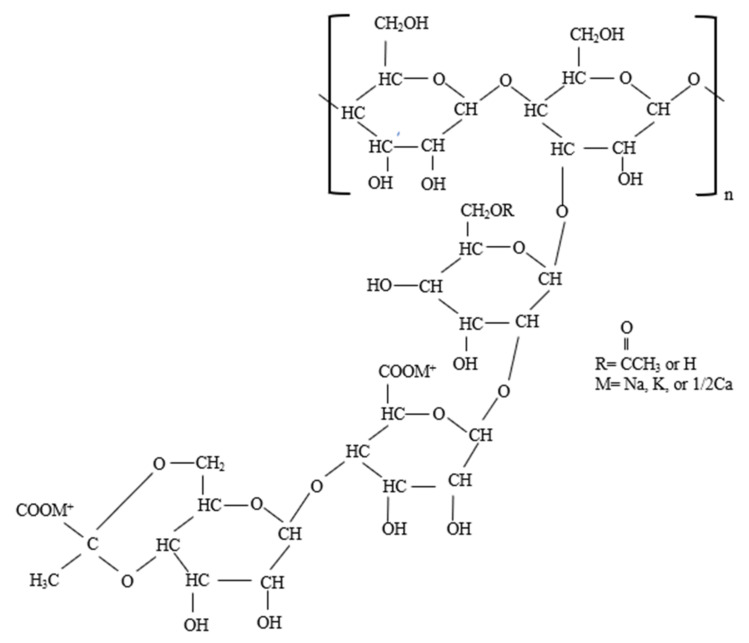
Structure of xanthan gum [17].

Xanthan gum is a nontoxic biodegradable polysaccharide produced by the action of several bacteria on glucose or its isomer fructose. The most commonly used bacteria for the fermentation process is *Xanthomonas campestris*. Figure 3 illustrates the chemical structure of xanthan gum showing its monomers of glucose, mannose, and glucuronic units. Moreover, the polymer contains acetate and pyruvate groups in its side chain. The high molecular weight of xanthan gum polymer accounts for its thickening ability. Furthermore, polymer chains are rigid, which makes them resistant to mechanical shear, high salinity, and/or divalent ion concentration.

As compared to HPAM, xanthan gum is relatively more stable in harsh reservoir conditions. In an aqueous solution, xanthan displays an order-to-disorder conformation. On the other hand, the presence of ionic concentration makes its macromolecular structure transit from a disordered conformation to a more rigid or ordered structure due to the charge screening effect. Zhong et al. [28] evaluated the impact of solution ionic strength on the viscous property of xanthan gum at varying polymer concentrations. At low polymer concentration (600 mg/L), the inorganic cations reduced the viscous property of the polymer. The effect of divalent ion concentration (Ca^2+^) was more pronounced than that of monovalent cation (Na^+^) [29]. However, at higher polymer concentrations, the viscosity of xanthan gum increased with an increase in inorganic cation concentration. The authors noted that at 5000 mg/L of xanthan gum, the introduction of 200, 500, and 1000 mg/L Ca^2+^ ions increased the viscosity of xanthan gum solution by 475% [28]. Meanwhile, the thermal stability of xanthan gum is dependent on the salinity of the aqueous solution. The xanthan solution is thermally stable when the polymer structure is ordered (at high ionic concentration) and unstable when the macromolecular structure is disordered (at low ionic concentration) [19].

Xanthan gum exhibits non-Newtonian behavior, and its behavior under shear is often analyzed with Ostwald and Herschel–Bulkley models [30]. The polymer exhibits high viscosity behavior at a low shear rate due to its macromolecular aggregation resulting from the presence of hydrogen bonding and polymer entanglements. However, the polymer viscosity decreases as the shear rate increases, displaying a shear thinning behavior which corresponds to appropriate injectivity for field operations. The pseudoplastic behavior of the polymer at a high shear rate is attributed to the orientation of the polymeric chains, which disentangle and disperse the macromolecular aggregate, along the line of the flow [30,31].

#### 3.1.2. Cellulose

**Figure 4:**
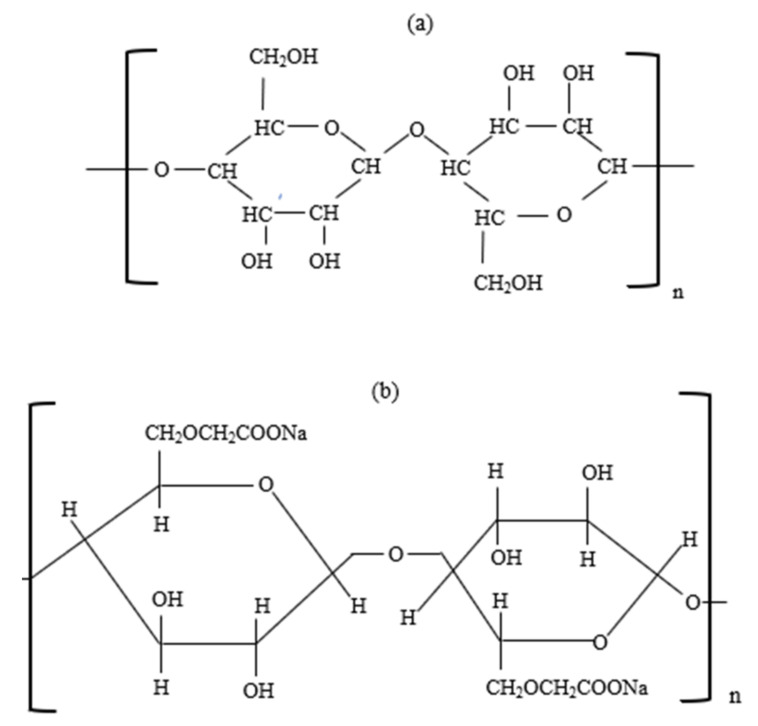

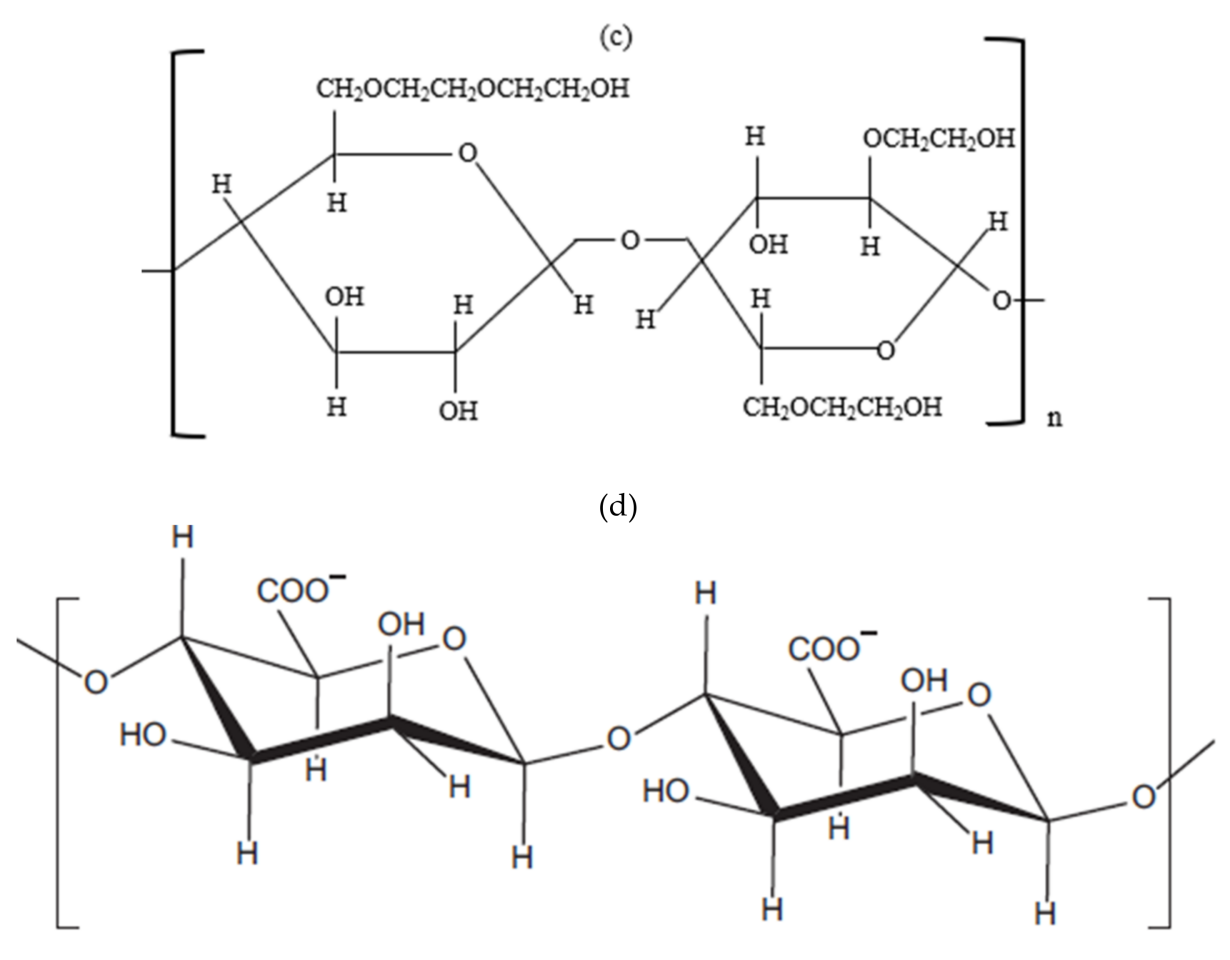
(**a**) Cellulose, (**b**) carboxymethylcellulose, (**c**) hydroxyethylcellulose, and (**d**) nanocellulose [16,17,34].

Cellulose is usually derived from the tissue of plant cell walls and eukaryotic cells and is widely regarded as the most abundant biopolymer in the world. This universal biopolymer can be found in bamboo, cotton, wood, and sometimes bacteria. Cellulose is described by the molecular formula ${\left(C_{6}H_{10}O_{5}\right)}_{n}$, where n is the degree of polymerization. This natural polymer is connected by *β*-(1,4) glycosidic bonds and is depicted in Figure 4. The structure distribution determines the properties of cellulose. Cellulose can withstand high mechanical shearing and temperature due to its network structure [32]. Nonetheless, the network structure causes heterogeneous swelling and insolubility. To meet the requirement of the petroleum industry, the surface of the cellulosic polymers is more commonly modified [33]. Several types of cellulose have been exploited for EOR. These include hydroxyethylcellulose, carboxymethylcellulose, and nanocellulose.

Hydroxyethylcellulose is an environmentally friendly nonionic cellulose derivative obtained by the chemical modification of insoluble cellulose. This polymer is tolerant to salinity, temperature, and mechanical shearing due to its rigid polymer chain structure. A major concern for hydroxyethylcellulose is its instability at low pH due to hydrolysis of the acetal linkages on the polymer backbone, but the polymer exhibits good stability at neutral and high pH. Other major concerns associated with hydroxyethylcellulose are oxidation and enzymatic degradation. Recently, hydrophobically modified hydroxyethylcellulose derived by hydrophobic modification of the macromolecular chain has been synthesized and exhibited sterling properties suitable for EOR. The intermolecular interaction between the hydrophobic moieties and the polymer backbone results in excellent rheological properties [35]. Liu et al. [36] modified the surface of hydroxyethylcellulose with bromododecane and investigated the rheological properties. The synthesized polymer shows improved viscous and elastic properties and good resistance to salinity, temperature, shear, and acid/alkali.

Carboxymethylcellulose is a derivative of cellulose formed by reacting the insoluble cellulose with chloroacetic acid in the presence of an alkaline medium (see Figure 4b). Carboxymethylcellulose has a varying structure which depends on the degree of substitution of the hydroxyl groups on the anhydroglucose linkages. The degree of substitution of ${\left(C_{6}H_{10}O_{5}\right)}_{n}$ and the distribution of carboxymethyl substituents dictates the properties of carboxymethylcellulose [16]. For example, the substitution of the hydroxyl group on the surface of the polymer with an alkali metal makes the polymer becomes soluble in water. Like other polymers, the rheological and viscoelastic properties of carboxymethyl-cellulose are dictated by the polymer concentration. Carboxymethyl cellulose displays elastic properties when the prevailing polymer concentration in the solution is greater than the critical concentration and shows viscous properties when the concentration is lower than the critical concentration [17].

Nanocellulose arose from recent development in nanotechnology which involves developing materials with at least one dimension on the nano scale (1–100 nm). Due to its nanofibrillar structure, cellulose is an ideal nanomaterial candidate. Nanocellulose has high functionality owing to its unique properties such as template structure, low density, large surface area, good modifiability, and biodegradability [33]. It is categorized into three types, namely cellulose nanocrystals, cellulose nanofibrils, and bacterial nanocellulose (see Table 1), according to Li et al. [37]. Owing to the abundant hydroxyl groups on its surface, nanocellulose is soluble in polar solvents. Besides, its colloid and interfacial behavior can be easily modified to increase its hydrophobicity by adsorption of different charged compounds on its surface.

Li et al. [38] synthesized nanocellulose and evaluated its properties for EOR. The synthesized nanocellulose was grafted with 2-acrylamido-2-methylpropane sulfonic acid (AMPS) and hydrophobic groups. The synthesized nanocellulose exhibited superior salt tolerance and salt thickening behavior due to the incorporation of hydrophobic groups. More importantly, the nanoscale structure permits its penetration in low-permeability and low-porosity reservoirs. The viscosity of nanocellulose decrease with an increase in temperature. Moreover, it exhibits pseudoplastic behavior in the dilute region and thixotropic behavior in the semidilute region [39].

#### 3.1.3. Guar Gum

**Figure 5:**
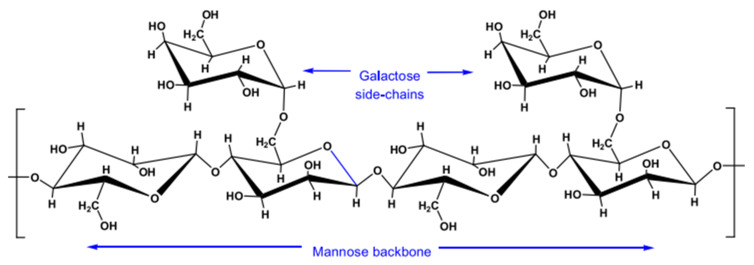
Molecular structure of guar gum [41].

Guar gum is a hydrophilic biopolymer derived from the endosperm of leguminous plants of *Cyamopsis psoraloides* and *Cyamopsis tetragonolobus*. As illustrated in Figure 5, guar gum is made up of linear backbone chains of (1–4)-*β*-d-mannopyranosyl principal units and (1–6)-*α*-d-galactopyranosyl branch units linked to the principal chain. Guar gum is soluble in polar solvents but insoluble in organic solvents. Guar gum possesses good hydration properties. A low concentration of guar gum yields high viscosity at a low shear rate because it possesses a large hydrodynamic volume and intermolecular interaction. As the shear rate increases, the polymer exhibits shear thinning behavior [40]. The viscosity of guar gum polymer increases in the presence of solution salinity. However, divalent cations effectively screen the polymer and cause it to precipitate at high concentrations. Guar gum is insoluble at low temperatures; hence, the polymer viscosity increases. Nonetheless, at high temperatures, the viscosity of the polymer decreases. Finally, guar gum presents a high risk of plugging because it is not completely hydrated.

#### 3.1.4. Welan Gum

**Figure 6:**
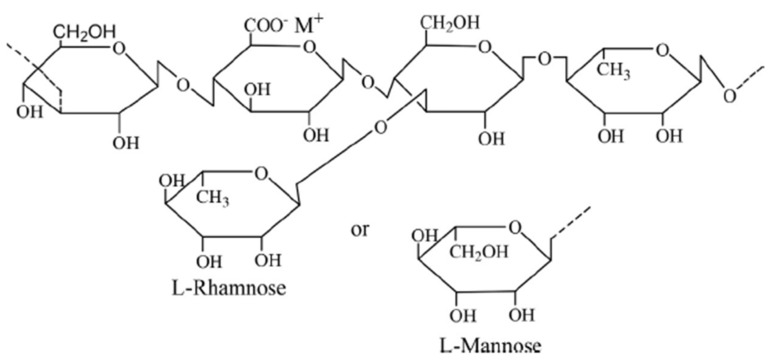
Molecular structure of welan gum [16].

Welan gum is a nongelling anionic polysaccharide secreted by the fermentation of sugar with Alcaligenes specie bacteria and made up of a pentasaccharide repeating unit [42,43]. As depicted in Figure 6, the repeating units are *β*-1,3-d-glucopyranosyl, *β*-1,4-d-glucuronopyranosyl, *β*-1,4-d-glucopyranosyl, *α*-1,4-l-rhamnopyranosyl, and a single monosaccharide side chain at o-3 of the 4-linked glucopyranosyl. The repeating units are characterized by acetyl and glyceryl substituents. One-third of the monosaccharide side chain linkage contains *α*-l-mannopyranosyl groups while the remainder contains *α*-l-rhamnopyranosyl groups [16,29].

As compared to a xanthan gum solution of the same molecular weight, the viscosity of welan gum is higher in an aqueous solution. This is attributed to the chain arrangement of the three-fold double-helix structure of welan gum [29]. However, due to the anionic charges on the polymer backbone, the viscosity, and viscoelastic properties of welan gum solution are affected by the presence of inorganic cations (Na^+^ and Ca^2+^). The ionic charges of the inorganic cations screen the polyelectrolyte and cause the shrinkage and coiling of the macromolecular chains of the polymer. Besides, in high-temperature conditions, chain decomposition of the polymer occurs and leads to a slight decrease in solution viscosity, especially at a low shear rate. The glyceryl groups of welan gum result in the formation of a double-helical conformation which is responsible for the viscosity at high temperatures. Welan gum has a better salt and temperature tolerance than xanthan gum due to its configuration [44].

Welan gum exhibits pseudoplastic behavior at a low shear rate. The shear thinning behavior of the polymer is due to the orientation of its macromolecular chain along the line of flow. At a low shear rate, the polymer stretches and intertwines to form aggregates that resist flow, thereby resulting in high viscosity. Contrariwise, as the shear rate increases, the aggregates disentangle and disperse along the direction of flow; consequently, the viscosity of the polymer solution decreases [45].

#### 3.1.5. Schizophyllan

**Figure 7:**
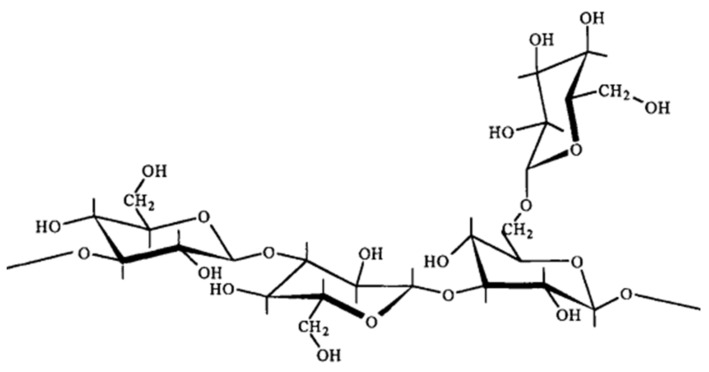
Molecular structure of schizophyllan [16].

Schizophyllan is a nonionic biopolymer extracted from fungus *Shizophyllum* via a fermentation process using glucose as the carbon source [46]. As shown in Figure 7, the polymer comprises linearly linked *β*-(1,3)-d-glucose residues with one *β*-(1,6)-d-glucose for every three main chain residues [16]. The excellent physiochemical properties of schizophyllan polymeric solution are due to its inherent stiff triple-helical conformation and intermolecular interaction resulting from the presence of hydrogen bonding [47]. In fact, this polymer has high salinity and temperature tolerance. Additionally, the polymer exhibits shear thinning behavior in the presence of shear forces.

### 3.2. Synthetic Polymers

Several synthetic polymers exist in the literature and have been exploited for EOR in the laboratory. The synthetic polymers are commonly categorized into polyacrylamide (PAM), hydrolyzed polyacrylamide (HPAM), and hydrophobically associating polyacrylamide (HAPAM).

#### 3.2.1. Polyacrylamide (PAM)

**Figure 8:**
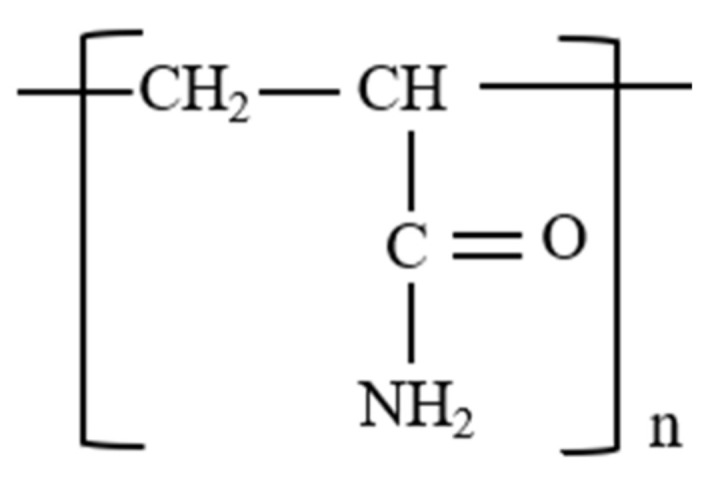
Molecular structure of PAM [20].

Polyacrylamide is a renowned thickening agent for EOR applications. This is because of its high molecular weight (>1 × 10^6^ g/mol). In its unhydrolyzed form, PAM is nonionic in nature (see Figure 8). Hence, high adsorption of the polymer on mineral surfaces is prevalent. This limits its direct application for chemical EOR. Nonetheless, due to the inherent properties of the polymer, it is mostly used in the hydrolyzed form. Several modifications of PAM that yield lower adsorption and better physicochemical properties desired for EOR have been performed and utilized.

#### 3.2.2. HPAM

**Figure 9:**
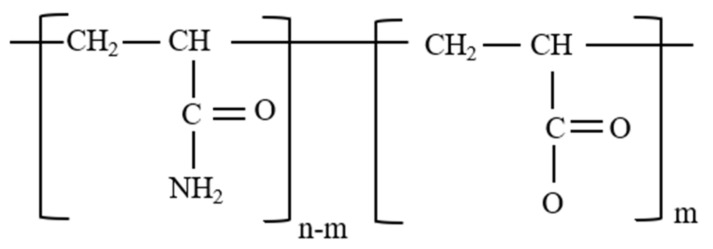
Molecular structure of HPAM [20].

HPAM is the most widely used polymer for field application of polymer floods because it can tolerate high mechanical forces present when flooding a reservoir. Besides, HPAM is resistant to bacterial attack and is a low-cost polymer. This polymer is synthesized from the copolymerization of sodium acrylate with acrylamide or partial hydrolysis of polyacrylamide and polyacrylic acid and is depicted in Figure 9 [20]. When dissolved in water, the polymer stretches due to electrostatic charges on the polymer backbone, and the viscosity of the polymeric solution increases. Factors that influence the viscous property of HPAM are the molecular weight of the polymer, concentration of the polymer, degree of hydrolysis, salinity, temperature, and shear rate [48].

Lower-molecular-weight HPAM has lower viscosity as compared to high-molecular-weight HPAM characterized by high viscosity and elasticity [49]. Furthermore, an increase in the concentration of HPAM causes an increase in the viscosity of the polymeric solution. The optimal degree of hydrolysis (DOH) of acrylamide is 25–35%. At lower DOH, the polymer is insoluble. Meanwhile, higher DOH causes the polymer to become sensitive to brine salinity and hardness and lose its viscous properties [21]. The thickening capability of HPAM is reduced in the presence of brines. This is attributed to the screening effect of the cations on the polymer backbone which causes a reduction in its electrostatic repulsion and consequently a lower hydrodynamic volume of the polymer [50]. Divalent cations have a more destructive effect on HPAM as compared to monovalent cations. Besides, the viscosity of HPAM shows a strong dependence on the temperature condition of the solution. As temperature increases, the viscosity of the HPAM solution decreases due to the thermal motion of the polymeric chains which causes intermolecular interaction of the macromolecule to decrease [51]. HPAM exhibits shear thinning/pseudoplastic and shear thickening behavior in the presence of shear.

#### 3.2.3. HAPAM

Due to the limitations of PAM and HPAM, HAPAM was developed as a derivative of acrylamide-based polymers by introducing comonomers into the polymer backbone. Comonomer additives are added to contribute to the molecular weight of the polymer. They improve the rheological and stability properties of the polymer in high-temperature and high-salinity conditions. Several salt- and temperature-tolerant comonomers have been exploited for HAPAM. Hence, HAPAM has better mobility reduction and higher incremental oil recovery when used for oil displacement. The incremental oil recovery of HAPAM has been attributed to the effect of elastic turbulence induced by the intermolecular association of hydrophobic comonomers during flow in porous media [52]. The performance of HAPAM is characterized by the critical aggregation concentration (CAC) of the polymer. The CAC is the inflection point or the threshold concentration that characterizes the behavior of the polymer. Below the CAC, the rheology of the polymer is low due to intramolecular interactions between the polymer chains. Conversely, above the CAC, enhanced rheological properties of the polymer occur due to intermolecular interactions between the polymer chains [53].

Despite its higher efficiency in numerous laboratory experiments, full field implementation of HAPAM remains limited. This may be because the functionality of the synthesized HAPAM is dictated by the type and nature of the comonomer used in its synthesis.

More importantly, careful selection of comonomers is required because their efficiency is dependent on the method of preparation and/or critical reservoir parameters such as salinity and temperature, which makes the overall process complex. Under increased salinity and divalent ion concentration, HAPAM exhibits different rheological behavior that is dependent on the polymer concentration, molecular structure of HAPAM, and type of hydrophobe.

Sarsenbekuly et al. [54] synthesized a novel low-molecular-weight HAPAM and measured the viscosity performance information water salinity [54]. The rheology of the polymer showed a nonmonotonic trend. Initially, the polymer viscosity decreases with increasing salinity until it reaches 20,000 mg/L NaCl concentration. The decrease in polymer viscosity was attributed to a reduction in repulsion and compression of the macromolecular chain resulting from the hydration effects of the electrolytes on the ionic group of the copolymer. Above this NaCl concentration, the viscosity of the polymer increases with increasing salinity until it reaches 80,000 mg/L. The sudden increase in polymer viscosity is due to the hydrophobic associative effect. The increased salt concentration causes enhancement in the degree of association of the polymer by lowering the solubility of the hydrophobic moieties, thus causing the formation of intermolecular aggregates and an increase in the hydrodynamic volume of the polymer with water. Quan et al. [55] observed similar properties with amphoteric HAPAM synthesized from *N*,*N*-dimethyl octadecyl allyl ammonium chloride and sodium-4-styrenesulfonate monomers.

To investigate the effect of the preparation method on the properties of HAPAM, Maia et al. [56] synthesized HAPAM by micellar copolymerization of acrylamide with dihexylacrylamide and performed characterization using nuclear magnetic resonance (NMR) and dynamic light scattering (DLS). The rheological behavior of the synthesized polymer under varying salinity concentrations was evaluated under different preparation conditions. Firstly, the synthesized polymer powder (0.5 g/L) was added to a saline solution (concentration 0–100 g/L). Under this condition, the viscosity of the polymer is reduced as the concentration of NaCl increases. This was attributed to the screening effect of the cation on the charged polymer moieties leading to intramolecular association. Subsequently, the rheological behavior of HAPAM was studied by adding salt powder to polymer solution. Using this method, the viscosity initially increases as the salt concentration increases until it reaches a maximum value around 60 g/L NaCl concentration; thereafter, the viscosity decreases. Lastly, the rheology of HAPAM was evaluated by adding polymer solution to varying saline solution concentrations. Under this preparation condition, the viscosity of the polymeric solution increases with increase in salinity, which is ascribed to the easiness of interaction between the polymer chain and the salt solution.

The effect of temperature on the rheological properties of the HAPAM depends on the concentration regime. When the polymer concentration is lower than the CAC, a decrease in the viscosity of the polymer is recorded with an increase in temperature. Sarsenbekuly et al. [54] observed that the viscosity of synthesized polymeric solution decreased with an increase in temperature for lower concentrations of HAPAM [54]. Likewise, Yang et al. [57] noted that the addition of a lower concentration (10%) of *N*-vinyl-2-pyrrolidone hydrophobic moieties to a copolymer of acrylamide and acrylic acid yielded a lower viscosity of the polymer. On the other hand, when the polymer concentration is above the CAC, the viscosity of the polymer increases with temperature until it reaches a maximum point and thereafter decreases. At higher temperatures, the macromolecular chain of the polymer is broken, which ultimately accelerates polymer decomposition [58]. Quan et al. [55] investigated the effect of temperature on synthesized amphoteric HAPAM and ascribed the initial viscosity increment versus temperature to the formation of intermolecular hydrophobic aggregates. Besides, hydrophobic interaction is an endothermic entropy-driven process [53,59]. Subsequently, the viscosity of the solution decreases with an increase in temperature after reaching the maximum, which was attributed to the destruction of the protective structure around the hydrophobic group which led to rapid molecular motions and consequently weakened the hydrophobic effect. A similar property of HAPAM was observed by Shi et al. [59] using hydrophobic monomer hexadecyl-allyl-dimethyl ammonium chloride with a copolymer of acrylamide (AM) and acrylic acid (AA).

## 4. Polymer Flooding

The application of polymers for EOR has demonstrated excellent recovery rates for medium, heavy, and extra-heavy oil. Hence, numerous experimental, pilot and field-scale applications of polymers for chemical EOR exist in the literature. Nonetheless, most of the field applications of polymer flooding have been limited to sandstone formations. This may be because of the complexities associated with carbonates such as vugs, fractures, and heterogeneities. The success of polymer flooding EOR projects depends on reservoir rock and fluid properties. These include lithology, location (onshore or offshore), depth, porosity, permeability, heterogeneity, oil viscosity, temperature, salinity, hardness, oil saturation, oil mobility, polymer type, and slug properties [60]. Hence, screening criteria for polymer flooding projects have been developed by several studies and are presented in Table 1. Table 2 presents the merits and demerits of EOR polymers, while Table 3 shows some experimental studies of polymer flooding EOR. More details on the field application of polymer flooding have been summarized by [19,61].

## 5. Binary Combination of Polymers and Other Additives for EOR

### 5.1. Polymer Foam Flooding

Gas injection is one of the earliest employed EOR schemes. The hydrocarbon and/or non-hydrocarbon gases (e.g., methane, air, nitrogen, and carbon dioxide) are injected to flood the reservoir with residual oil [75]. Even though the injected gases are vapors at atmospheric pressure and temperature conditions, their properties may change to those of supercritical fluids at typical reservoir temperature and pressure [76]. The gas injection process is broadly classified into miscible and immiscible flooding. For miscible gas flooding, the gas is injected at and/or beyond the minimum miscibility pressure. The EOR mechanisms include the mass transfer of components between the oil resident in the reservoir and the injected gas, swelling, interfacial tension, and viscosity reduction of the oil phase. In the case of immiscible gas flooding, the injection of gas takes place below the minimum miscibility pressure, and hence, the reservoir pressure is maintained. Nonetheless, the use of gas flooding for EOR suffers from low areal and vertical sweep efficiencies. Besides, other issues such as gravity override, gas segregation, and channeling of gas via high permeability streaks reduce the efficiency of the injected gas [77,78]. To improve the mobility and overcome other limitations of injected gas, foamed-gas injection was developed and subsequently implemented for field application of gas EOR.

Foam in porous media is defined as gas dispersions in liquid wherein the liquid phase is continuous and a portion of the gas phase is made discontinuous by thin liquid films known as lamellae [79]. Foams are generated when a foaming agent that contains liquid is brought in contact with gases such as N_2_, CO_2_, and air and sufficient mechanical energy is supplied. The mechanisms of foam generation are classified into leave behind, snap off, and bubble division [80]. In the reservoir, the generated foam reduces the relative permeability of the gas phase and increases the apparent viscosity of the displacing fluid, thereby controlling gas mobility. The apparent viscosity of the displacing fluid is raised by drag forces placed on the pore walls by moving bubbles, while the relative permeability of the gas is reduced by gas trapping [77]. In a heterogeneous reservoir, foams aid diversion of subsequent injectant from thief zones to low-permeability regions of the reservoir. Despite the numerous advantages of foams for oil recovery in reservoirs, they are thermodynamically unstable, and rapid collapse of the lamellae occurs, diminishing their efficiency. The instability of foams is caused by three interdependent mechanisms known as drainage, coalescence, and coarsening [81]. To maximize the potential of foam for EOR, several surface-active agents have been explored to stabilize foams. These include surfactants, proteins, polymers, ionic liquids, and more recently nanoparticles [82,83,84,85].

**Figure 10:**
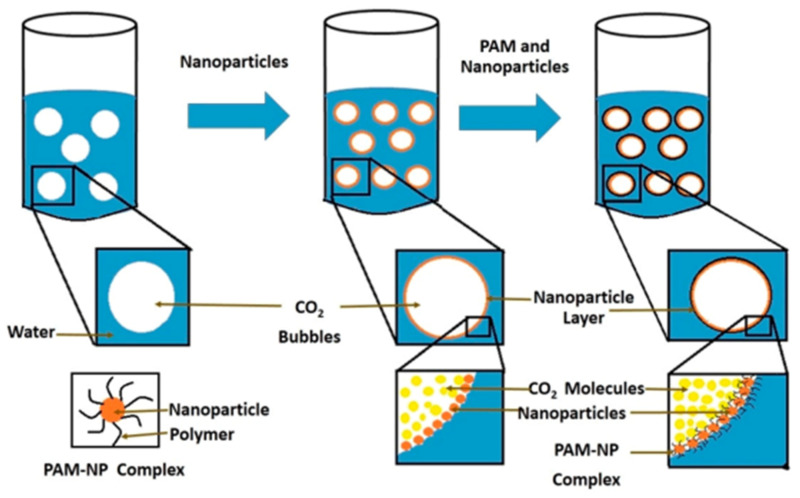
Schematics of polymer-stabilized nanoparticle foam [87].

Polymers have been explored explicitly for stabilizing foam due to their macromolecular structure and other intrinsic properties. Due to their inherent properties (e.g., viscoelasticity), only a small concentration of polymer is required to stabilize foam, making the overall process economical and cost-effective. The use of polymer increases the viscosity and stability of the foam and consequently minimizes the liquid drainage rate [86]. Therefore, polymer-stabilized foams display excellent mobility control properties when used for oil recovery. Additionally, polymers are used as an additive with surfactant-stabilized or nanoparticle-stabilized foams to prevent the desorption of the surfactant molecules and nanoparticles from the interface of the lamellae, thus preventing coalescence of the foam and improving its half-life (see Figure 10).

Synthetic polymers and biopolymers have shown sterling properties for stabilizing foam [88,89]. Wang et al. [90] examined the influence of HPAM on alpha olefin sulfonate (AOS) foam stability. The biggest foam volume and best foam stability were obtained at 0.1 wt.%. The presence of HPAM was found to increase the surface tension and the foam viscosity. To evaluate the impact of polymer type on the stability and viscous properties of foam, Hernando et al. [91] investigated the efficiency and transport properties of foams stabilized with associative polymers and PAM in porous media. The study found that the use of amphiphilic polymers results in stronger interactions with surfactants; hence, foams stabilized with amphiphilic polymers are better than those stabilized with PAM or bare surfactant. The authors observed exchanges in the bulk and at the interface between surfactant molecules and the amphiphilic polymers which are responsible for the propagation of a more stable foam with a slower kinetics and a higher pressure drop. Similarly, Ahmed et al. [92] compared the foaming performance of a new associative polymer (Superpusher B 192) to HPAM. The associative polymer exhibited a higher foam strength and a 2-fold increase in apparent viscosity of foam compared to HPAM-stabilized foam. Due to improved foam viscosity, the associative polymer enhanced the bulk solution’s rheological properties and improved its tolerance. On the other hand, the polymer-free foam of AOS shows a rapid liquid drainage and resultantly a fast foam decay. The associative polymer-stabilized foam recorded 28% incremental oil recovery compared to 14% incremental oil recovery recorded by polymer-free foam [93]. Hence, it can be deduced that the addition of a hydrophobic chain enhanced polymer performance for foaming applications [94].

Bashir et al. [95] investigated CO_2_ foam stability and viscosity of nanoparticle/polymer-enhanced foam at high-temperature and high-salinity conditions in the presence of an oleic phase. Fumed SiO_2_ nanoparticles and rice husk ash was used as the nanoparticles while xanthan gum was used as the polymer. They noted that increasing the molecular weight of the polymer and reducing the nanoparticle sizes resulted in high foam stability. The improved foam performance was attributed to the presence of polymers and nanoparticles which snap the oil into emulsion droplets that pass through the lamellae easily without unloading the surfactant solution [96]. Wei et al. [97] evaluated the synergic effect of xanthan gum and alkyl polyglycoside (APG) on foam stability in the presence of oil. They observed that the liquid film has a higher viscosifying capacity in the presence of the polysaccharide. Two mechanisms were proposed for the stability of the polymeric foams. Firstly, the presence of xanthan gum enhances the interfacial elasticity and forms a denser adsorption layer which improves the formation of pseudoemulsion films, thereby increasing the stability of the oil-laden foam. Lastly, the increase in liquid viscosity and emulsion stability inhibits liquid drainage. Wei et al. [98] studied the stability of foam in the presence of surface-grafted nanocellulose. The presence of the surface-grafted nanocellulose in the foam film inhibited liquid drainage and increased the thickness of the foam film. Zhang et al. [99] observed that the synergistic combination of lignin–cellulose nanofibrils and cationic/anionic surfactants generated robust foams and inhibited liquid drainage. Additionally, the composite of welan gum and hydroxylpropyl methylcellulose exhibited sterling properties in stabilizing foams due to its excellent shear thinning properties and physical interaction.

Apart from polymer type, other factors that influence the efficiency and effectiveness of polymer foams are the viscosity of the oleic phase, the salinity, and the temperature conditions. The higher the viscosity of the oleic phase, the higher the susceptibility of foam stabilized by polymers to destruction. Likewise, an increase in temperature portends destructive tendencies in the stability of foams, while an increase in salinity causes an increase in foam stability against liquid drainage and coalescence [100]. Dehdari et al. [89] studied the influence of oil type on the stability of foams stabilized by polyvinyl alcohol (PVA) in the presence of surfactants and nanoparticles. As compared to light oil, they noted that heavy oil destabilizes polymer foams more. Moreover, they observed that an increase in the aqueous phase salinity in the presence of PVA caused the foam stability to increase. Nonetheless, the polymer-stabilized foams have better stability in the presence of temperature. Fu and Liu [101] evaluated the salinity and thermal resistance of CO_2_ foam stabilized in the presence of nanoparticles, surfactants, and hydroxylethylcellulose polymers. They noted that the increase in temperature resulted in the decrease of the apparent viscosity of CO_2_ foams and accelerated the drainage of the interfacial film. However, the presence of the polymer enhanced the thermal resistance of the CO_2_ foams.

Overall, the use of polymers for enhancing foam has several benefits and advantages for EOR. They have demonstrated proven efficiency in the recovery of conventional and heavy oil in homogeneous, heterogeneous, and fractured reservoirs. Apart from oil production, recent research has suggested that polymer-enhanced foams have proven more beneficial for gas sequestration and storage than conventional foams [102]. For an efficient polymer foam flooding operation, an optimum selection of the properties of the polymer is required. The use of nanoparticles as additives for polymer-stabilized foam is also encouraged. Finally, some areas of contention exist among researchers on the foaming properties of polymer-stabilized foam, which need to be clarified. For example, some research stated that the molecular weight of foam has little or no influence on the foaming capability, while others opined that the increase in molecular weight of the polymer causes high foam stability [90,95].

### 5.2. Alkali–Polymer Flooding

Alkali–polymer flooding is a synergistic combination of the efficiency of an alkaline solution and a polymer flood to improve oil recovery. This chemical EOR arose due to the inefficiency of alkaline EOR flooding. Alkali injectants alter the fluid–fluid and rock–fluid properties such as oil–water interfacial tension and wettability of the porous media. More importantly, the alkali solution reacts with the naphthenic contents of the crude oil to generate in situ soap which forms a stable emulsion and ultralow IFT [103]. Nonetheless, alkalis lack the required mobility to push the oil bank, especially when applied in heavy oil reservoirs. The utilization of a polymer with the alkali helps provide the required mobility ratio for EOR.

The effect of the alkalis on the behavior of the polymer depends on the alkali concentration, pH, and polymer types. The resultant effect can be an increase in the ionic strength of the solution or pH [104]. When used with synthetic polyacrylamide, the alkali causes the acceleration of the degree of hydrolysis of the polymer. At low alkali concentration, the polymer molecules have low viscosity due to the tight coil conformation. As the concentration of the alkali increases, the pH of the solution increases and electric repulsion occurs along the polymer chains, causing a large hydrodynamic radius of the macromolecule and an increase in polymer solution viscosity. Chul et al. [105] observed an increase in the viscosity of HPAM with the addition of caustic alkali (NaOH) in the presence of brine and temperature. However, at high alkali concentrations, the polymer solution viscosity decreases. This is attributed to the increase in the ionic strength of the solution. The lower viscosity of the polymer at a high concentration of alkali may be desirable to improve polymer injectivity near the wellbore region in tight formations. The reintroduction of waterflooding will cause a reduction in the ionic strength and cause the polymer to increase in size. Subsequently, this causes an increase in resistance to flow and diversion to poorly swept regions, thereby improving the sweep efficiency. A similar result was observed for the effect of alkali on a biopolymer (xanthan gum) [30,106].

Additionally, the alkali causes an increase in the negative charge repulsion between the rock surface and the polymer molecule. Consequently, a decrease in the adsorption of the polymer on the rock surface occurs. Moreover, the presence of the polymer reduces the consumption of the alkali compared to when using the alkali solution alone. Ding et al. [107] performed a mechanistic study of alkali–polymer flood for a reservoir characterized by heavy oil. The alkali–polymer system formed water-in-oil emulsions and achieved ultralow IFT values. In high water saturation zones, the water-in-oil emulsions formed during alkali–polymer flooding increased the resistance to water flow, thereby improving the sweep efficiency. The alkali–polymer flooding yielded 40.2% incremental oil recovery for a heavy oil (oil viscosity = 1300 cP) reservoir. Overall, the flooding tests show that the use of alkali–polymer floods is more efficient than the use of bare alkali flooding or polymer flooding [108]. Despite the previous studies, the fluid–fluid and rock–fluid properties of alkali–polymer flooding are not properly elucidated in the literature. Additionally, scaling problems associated with the use of alkalis pose a huge challenge for field implementation of alkali–polymer floods. Finally, for maximum efficiency, an optimum concentration of alkali and polymer slug should be determined based on the rock and fluid properties in the reservoir.

### 5.3. Surfactant–Polymer Flooding

Surfactants with their hydrophobic groups can only improve the pore-scale displacement efficiency by lowering the IFT, altering wettability, and stabilizing emulsions [84]. Hence, surfactant flooding is only suitable for recovering capillary-trapped oils and may not achieve the desired efficiency in heavy oil reservoirs. On the other hand, the use of polymer increases the viscosity of the injectant, improves the mobility ratio, and consequently improves the volumetric sweep efficiency. Polymers do not cause significant changes at the microscopic level. Meanwhile, the overall recovery efficiency is a combination of microscopic and macroscopic efficiencies. Hence, the synergic application of surfactant and polymer flooding is used to overcome the deficiency of the individual chemical and boost oil recovery [36].

Nonetheless, a careful selection of chemicals is required to achieve the desired efficiency during the surfactant–polymer flooding process. This is because the combination of a surfactant and a polymer with widely different properties can cause phase separation [109]. Furthermore, the injection slug of the SP flooding process depends on the aim of the flooding process. Due to competitive adsorption of the chemicals in porous media, the first injectant can act as a sacrificial agent by reducing the pore space available for the subsequently injected chemical while at the same time contributing to the recovery process. When a polymer is injected as the primary slug, it inhibits the adsorption of surfactant and ensures conformance control. On the other hand, the use of a polymer as a secondary slug helps to sweep the bypassed oil occasioned by the viscous fingering phenomena encountered during the water and surfactant flooding process [65]. Even though both chemicals are not injected at the same time, the interaction between the surfactant and polymer should be considered when developing the screening criteria because the mixing of the chemicals may occur via diffusion and dispersion phenomena.

**Figure 11:**
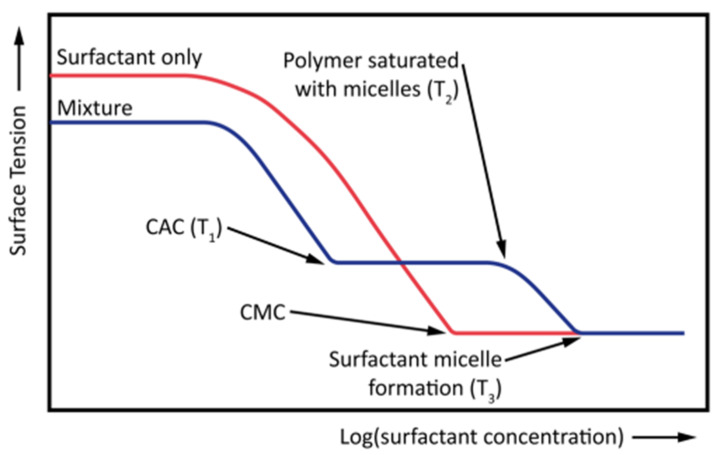
Polymer effect on IFT [111].

Several studies have reported the effects of surfactants on polymer behavior and vice versa [52,110]. The measurement of IFT as a function of polymer and surfactant concentrations is one of the most visible outcomes of this interaction. When the polymer is added to the system, two different concentration values are observed to occur and replace the CMC of the system, as depicted in Figure 11. The first concentration is the critical aggregation concentration (CAC), which is smaller than the CMC, and the second concentration is the polymer saturation concentration, witnessed at a higher level than the CMC. The former is characterized by the adsorption of surfactant molecules and interaction with polymer chains, while the latter is characterized by surfactant molecules forming micelles with the polymer molecules present in the solution [111].

Depending upon the charges on the surfactant and polymer investigated, the interactions are usually ascribed to electrostatic or hydrophobic effects. Afolabi [52] investigated the effect of sodium dodecyl sulfate (SDS) on the rheological behavior of poly(acrylamide-co-*N*-dodecylacrylamide). The study reported an increase in viscosity of the polymer with increasing surfactant concentration until the surfactant reaches its CMC and subsequently the polymer viscosity decreases. This was attributed to hydrophobic interactions between the surfactant and the polymer. Similarly, Yusuf et al. [112] studied the effect of sodium dodecyl benzene sulfonate on the rheological, emulsion, and wettability alteration properties of carboxy methyl cellulose (CMC). They reported an increase in viscosity of the surfactant–polymer mixture until it reaches the CMC, above which the viscosity of the polymer decreases. They opined that hydrophobic microdomains of the surfactant at high concentration disrupt the intermolecular forces of the surfactant–polymer mixture, thereby causing a decrease in viscosity.

Furthermore, Kalam et al. [113] investigated the effect of spacer nature and counterions of a novel polyoxyethylene cationic surfactant on the rheological properties of cationic polyacrylamide polymers. They found that increasing surfactant concentration causes a reduction in the shear viscosity and elasticity of the surfactant–polymer mixture. Moreover, increasing temperature also caused a decrease in the storage and complex viscosity. On the other hand, they noted that the addition of phenyl ring in the spacer of the Gemini surfactant caused an increment in the viscosity and storage modulus of the surfactant–polymer system. As compared to bromide counterions, chloride counterions performed better in improving the rheological properties of the polymer. Ge et al. [114] examined the influence of betaine surfactant structure on the rheological properties of the surfactant–polymer (SP) mixture. Due to the electrostatic shielding effect, short-chain betaine surfactant was detrimental to the viscosity of the polymer solution. However, at high concentrations, long-chain betaine surfactant has a positive effect on the viscosity of the surfactant–polymer flooding process.

To reduce the quantity of chemicals injected, recent studies have developed polymeric surfactants from the fusion of an amphiphilic surfactant and the macromolecules of a polymer into one single chemical component. The newly synthesized chemicals also demonstrate the ability to alter the fluid–fluid and rock–fluid properties due to their surface-active nature. Although polymeric surfactant does not reduce the IFT to ultralow values like conventional surfactant, the IFT reduction (*~*10^−1^ mN/m) is considerably low enough to generate stable microemulsions [115]. Additionally, polymeric surfactants demonstrate sterling rheological properties and reduce the mobility of the injectant. Moreover, they exhibit shear thinning behavior at high shear rates, a process that is desirable in field application to avoid injectivity problems. Overall, a polymeric surfactant combines the interfacial property of a conventional surfactant and the viscoelastic properties of the polymer molecules [116].

As compared to conventional polymers, a polymeric surfactant exhibits better solution properties because of the presence of hydrophobic units in its molecular chain. Moreover, its molecules are joined together by hydrogen and van der Waals forces in its functional groups; hence, they develop tensile bonds that cause increased bulk viscosity and viscoelastic properties. Kumar et al. [117] studied the rheological properties of an anionic polymeric surfactant derived from Jatropha. The viscosity of the synthesized surfactant increases as the concentration of the surfactant increases, and they demonstrated good pseudoplastic behavior as the shear rate increases. Babu et al. [118] synthesized polymeric surfactant from castor oil and evaluated the rheological properties. The novel polymeric surfactant exhibited non-Newtonian behavior at a high shear rate with viscosity in the range of 10–40 cP, which is considerably higher than that of conventional surfactants. Pal et al. [119] evaluated the rheological properties of synthesized polymeric methyl ester sulfonate for the EOR process. Increasing the concentration of the polymeric surfactant caused an increase in the storage and loss modulus of the solution.

In addition to its good rheological properties, the polymeric surfactant showed good IFT and wettability reduction properties. Kumar et al. [117] noted that 6 g/L of polymeric surfactant reduced the IFT by 10-fold from 22.4 mN/m to 2.4 mN/m and altered the wettability of oil-wet quartz surface to water-wetting condition. Babu et al. [118] reported that polymeric surfactant from castor oil reduced the contact angle to less than 20° after 720 s. Mehrabianfar et al. [120] estimated the surface-active properties of a polymeric surfactant synthesized from an *Acanthephyllum* plant. The polymeric surfactant reduced the contact angle of oil-wet carbonate rock from 146° to 64°. Co et al. [121] noted that 2000 ppm of functionalized polymeric surfactant reduced the IFT of the oil–water interface to 0.15 mN/m. Besides, the presence of the functionalized polymeric surfactant caused the formation of water-in-oil emulsion with favorable properties for oil recovery when used with oil from the Illinois basin. Nowrouzi et al. [122] evaluated the IFT and EOR behavior of polymeric surfactant synthesized from Tragacanth gum at high-temperature conditions. The concentration of 2000 ppm of the polymeric surfactant reduced the IFT of the oil–water interface from 25.145 mN/m to 2.329 mN/m in 0.02 wt.% diluted formation water containing NaCl brine. Li et al. [123] examined the IFT properties of polymeric surfactant synthesized by micellar polymerization of sodium allyl-sulfonate, acrylic ester, acrylamide, and allyl glycidyl ether. As the concentration of the polymeric surfactant increases, intramolecular interaction with the polymeric surfactant gradually changes to intermolecular interaction, and the IFT of the oil–water interface is lowered from 32 mN/m to ~0.6 mN/m. This was ascribed to the adsorption of the hydrophobic chain of the polymeric surfactant at the oil–water interface. Moreover, the emulsion stability of the polymeric surfactant was compared to the SP system. The emulsion formed by the conventional SP system was completely stratified after 5 days, while the emulsion stabilized by the polymeric surfactant was stable and display no obvious stratification for a longer time.

SP and polymeric surfactant flooding display good oil recovery in sandstone and carbonate reservoir cores. Laboratory results show that the application of SP and polymeric surfactant flooding is also suitable for heavy oil reservoirs. Han et al. [124] examined the efficiency of high-performance SP formulation by mixing alpha olefin sulfonate (AOS), alcohol propoxysulfates, alkyl benzene sulfonate, and cocamidopropyl hydroxysultaine. Approximately 35% OOIP incremental oil recovery was achieved with 0.3 PV of NaCl-SP slug and 1 PV NaCl preflush. Li et al. [123] recorded 17.5% incremental oil recovery over waterflooding by using a novel synthesized polymeric surfactant. A summary of experimental results on SP and polymeric surfactant flooding is provided in Table 4. Additionally, field application of SP flooding has been reported with varying degrees of success [110,125]. For example, the application of SP flooding in the Shengli oilfield of China achieved better recovery than polymer flooding. When SP chemical was injected, the fluid entry profile was adjusted because the pressure of the injection well increased, thus enhancing the performance of profile control. Moreover, residual oil was displaced due to ultralow IFT. More importantly, SP flooding demonstrated excellent ability in reducing the water cut. The important critical parameters for the SP flooding process were identified as connectivity between production and injection wells, the volume of the injected chemicals, and the inherent properties (e.g., stability and compatibility) of the SP system [126].

### 5.4. Alkali–Surfactant–Polymer (ASP) Flooding

ASP flooding is the synergic combination of the efficiency of alkali, surfactant, and polymer blends to achieve incremental oil recovery. The presence of alkali and surfactant blends improves the pore-scale displacement efficiency by lowering the IFT of capillary-trapped oil and residual oil in the reservoir [20]. Besides, they alter the wettability of the porous media to a water-wetting condition desired to improve productivity. Meanwhile, the presence of the polymer blend enhances the macroscopic sweep efficiency, which is especially required for heavy oil [103]. On the other hand, the presence of the polymer reduces the water cut, and the overall oil recovery efficiency is depicted in Equation (2).

(2) $$E_{ro}=E_{do}E_{a}E_{v}\frac{S_{o}V_{p}}{B_{o}}\;\;\;$$

where *E_ro_* denotes the overall oil recovery efficiency, *E_do_* is the pore-scale displacement efficiency, *E_a_* is the areal sweep efficiency, *E_v_* is the vertical displacement efficiency, *S_o_* is the oil saturation, *V_p_* is the permeability variation, and *B_o_* is the oil formation volume factor.

**Figure 12:**
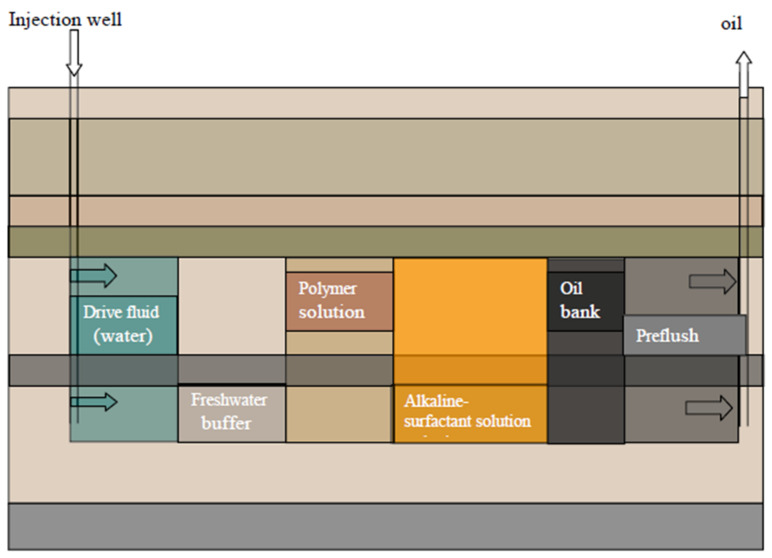
Schematic representation of ASP flooding [20].

An interplay of several mechanisms occurs during the ASP flooding process. Firstly, the alkali present in the injectant generates in situ soap [103]. The in situ generated soap has a low optimum salinity, whereas the injected surfactant is characterized by a relatively high optimum salinity. The mixture of the surfactant and the in situ generated soap ensures ultralow IFT values over a wide range of salinity [20]. Secondly, the presence of alkali in the injectant slug minimizes the adsorption of surfactant and polymer on the reservoir cores [132]. Furthermore, a stable emulsion is formed due to the in situ soap and surfactant. The presence of the polymer stabilizes the emulsion further due to its high viscosity which retards the emulsion against coalescence. Moreover, the synergic interaction between the surfactant and polymer reduces their adsorption to the pore spaces of the rock. Additionally, the polymer slug enhances the macroscopic sweep efficiency due to its viscosity and viscoelasticity [133]. The injection pattern is depicted in Figure 12. A preflush consisting of brine is first injected to tune the salinity or reservoir rock and fluid properties. Subsequently, a chemical slug of alkali and surfactant is injected and followed by a slug of polymer. Finally, a slug of chase water is used to optimize the recovery process. A synergic combination of the three chemicals causes the capillary number to increase and improves the mobility ratio, thereby increasing pore-scale displacement and sweep efficiency [134].

Several laboratory experiments confirmed the efficiency of ASP flooding in the recovery of oil from reservoirs. Sui et al. [135] investigated the efficiency of ASP coreflood of active oil at typical reservoir temperature and pressure of 62 °C and 1700 psig, respectively. The ASP flooding yielded a 44.5% incremental oil recovery over waterflooding. Moreover, Zhapbasbayev et al. [136] reported the ASP flooding of highly viscous oil with 407.4 cP and 300 cP in cores from the Eastern Moldabek field and Karahzanbas field of Russia. The ASP flooding yielded an incremental oil recovery of 19–37% over the waterflooding process. Furthermore, Panthi et al. [137] evaluated ASP flooding of viscous oil as a secondary and tertiary mode of oil recovery using sodium metaborate as the alkali, propoxy sulfate surfactant, and HPAM polymer. The use of ASP as a secondary and tertiary mode of oil recovery yielded an additional 47.8% and 44.9% oil recovery over conventional waterflood, respectively. Additionally, Fu et al. [138] observed an incremental oil recovery of 20% over waterflooding using organic alkali and petroleum sulfonate surfactant in an ASP flooding process. Liu et al. [139] noted that the use of ASP flooding in dolomite and silica sandpacks recovered 98% of the residual oil. Ghosh et al. [140] performed an experimental investigation of the application of ASP in a low-permeability tight carbonate reservoir and conducted modeling studies to understand geochemical interactions during the EOR process. Tertiary application of ASP flood resulted in the recovery of 77–87% OOIP. Panthi et al. [137] investigated the use of slug injection of ASP flood for heavy oil in a carbonate reservoir. The secondary surfactant flood reduced oil saturation to 3.1% and increased cumulative oil recovery to 95.6%.

Recently, to minimize toxicity associated with conventional surfactants when used in ASP flooding, several studies have synthesized natural and biosurfactants and evaluated their effectiveness in the ASP flooding process. Kesarwani et al. [141] synthesized a novel biodegradable surfactant from karanj oil and evaluated its efficiency in the ASP flooding process. An oil displacement test using sandpack flooding yielded an additional 32% incremental oil recovery. Nowrouzi et al. [142] synthesized a natural surfactant from the soapwort plant and evaluated its application in ASP injection slug for sandstone reservoirs. Ultralow IFT and wettability alteration of the sandstone core to the water-wetting condition were obtained. The slug combination of sodium hydroxide (NaOH), *soapwort* surfactant, and HPAM flood generated an incremental oil recovery of 32.1%. Nowrouzi et al. [143] utilized mucilage from the *hollyhocks* plant as a natural polymer and anionic surfactant synthesized from waste chicken fat and investigated their use for the ASP process in sandstone reservoirs. The ASP slug injection process increased oil recovery by 27.9%. Table 5 summarizes experimental studies of ASP flooding. Additionally, field application of ASP has been reported with varying degrees of success in Daqing oilfield of China, Taber South in Alberta, Tanner field, West Kiehl, Lawrence field, and Cambridge Minnelusa field of the USA.

Despite the field application of ASP for EOR, several challenges need to be addressed to ensure optimum application of this EOR technique. Firstly, the presence of the alkali at a high concentration may trigger the formation of scales near the wellbore or production system when it reacts with rock minerals [144]. Additionally, a high concentration of alkali causes hydrolysis of polymers and consequently reduces the viscoelasticity of the polymer. Meanwhile, polymer viscoelasticity is a prerequisite for achieving good sweep efficiency, especially in heterogeneous reservoirs [145]. Hence, an optimum concentration of alkali is required to be devised based on the process parameters (such as the formation type, clay type, and divalent cations) to achieve the desired efficiency. Moreover, the in situ generated soap, surfactant, and polymers generate stable oil-in-water emulsions which present unique separation challenges. Finally, there is a unique challenge presented by the treatment of produced water from the ASP flooding process due to the high concentration of oily and suspended solid contents [20].

### 5.5. Polymeric Nanofluid Flooding

Despite the efficiency of polymers in improving oil recovery, adsorption and retention of polymer macromolecules have been reported during chemical injection. Besides, the degradation (chemical, mechanical, and thermal) of polymer molecules reduces the efficiency during the polymer EOR process [153]. Recently, the use of additives to improve the physicochemical properties of polymers has been reported. Initially, the synthesis of temperature- and salt-tolerant polymers with good properties had been proposed but real field applications had been hampered by the economics and complexities of the polymers [19]. More recently, the incorporation of relatively inexpensive nanoparticles has been found to yield novel materials with fascinating properties for EOR. The nanoparticles and the polymer react via hydrogen bonding, electrostatic and van der Waals interaction, steric repulsion, and hydrophobic interaction [154]. The formed polymeric nanofluids exhibit salt-tolerant behavior, temperature tolerance, and high-performance characteristics. The incorporation of the nanoparticles in the polymer solution gives rise to an excellent rheological behavior of the polymer. Besides, the polymeric nanofluids exhibit lower adsorption and better stability in porous media, making them more efficient for recovering oil from the reservoir. Finally, they alter the fluid–fluid and rock–fluid properties [155].

**Figure 13:**
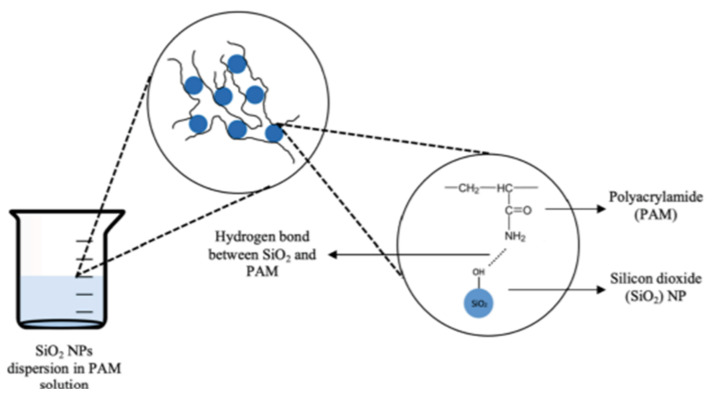
Bonding between nanoparticles and PAM [162].

**Figure 14:**
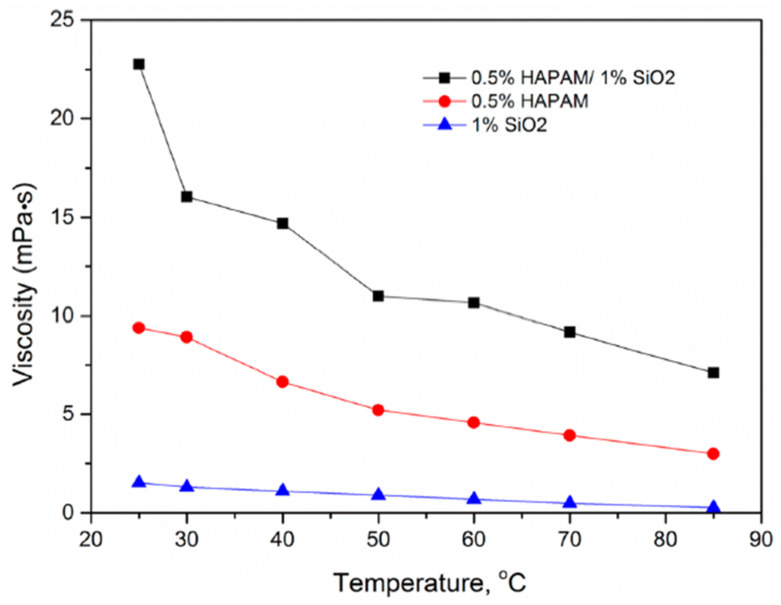
Viscosity behavior of PNF, HPAM, and SiO_2_ NPs (8 wt.% brine, shear rate 500–100 s^−1^) [159].

The improved rheological properties of polymeric nanofluids at high temperature and high salinity (HTHS) have been attributed to the formation of hydrogen bonds and the shielding effect of the nanoparticle on the polymer macromolecule (see Figure 13). Agi et al. [156] examined the rheological properties of PNF synthesized from starch by weak acid hydrolysis reaction and found that the synthesized PNF displayed better rheological properties than xanthan gum. Rezaei et al. [157] modified the surface of montmorillonite nanoclay and studied its rheological behavior with HPAM. The resultant polymer nanofluid displayed better rheological behavior, shear resistance, and higher oil recovery. Maurya and Mandal [158] investigated the rheological properties of polyacrylamide with SiO_2_ nanoparticles. They reported higher viscosity behavior of the PNF dispersion. Hu et al. [159] seeded acrylamide-based polymer with silica nanoparticles and studied the rheological and stability behavior under HTHS conditions. The presence of the SiO_2_ nanoparticles greatly improved the rheological and thermal stability behavior of the polymer, as illustrated in Figure 14. They noted that the presence of the nanoparticles leads to an increase in the viscosity of the polymer. This is ascribed to the formation of a three-dimensional network of floc bonded by hydrogen bonding which shields the polymer and minimizes degradation. In addition, Li et al. [34,38] observed that polymeric nanofluid of nanocellulose demonstrated excellent rheological properties. Similarly, Agi et al. [133] examined the rheological properties of PNF derived from okra mucilage and found it they exhibited good rheological properties in the presence of brine. Corredor-Rojas et al. [160,161] reported improved rheological properties, salt tolerance, thermal resistance, and shear resistance of polymeric nanofluid formed with modified silica nanoparticles and xanthan gum polymer.

Aside from the improved rheological behavior and salinity and thermal tolerance of polymeric nanofluids, the novel material demonstrated lower adsorption and better stability in porous media. Low adsorption and high stability are desired during chemical EOR. Bagaria et al. [163,164] studied the stability and adsorption properties of a polymeric nanofluid composed of iron oxide nanoparticles (IONPs) and an acrylamide-based polymer. The resultant polymeric nanofluid displayed lower adsorption and higher stability on silica sand surfaces due to steric repulsion. Cheraghian et al. [165] reported lower adsorption of polymeric nanofluids containing silica and clay nanoparticles on sandstone cores. Xue et al. [166] studied the stability properties of polymeric nanofluid made of IONPs and poly(AMPS-co-AA) copolymer under HTHS conditions. The polymeric nanofluid demonstrated good stability and transport properties in porous media. Iqbal et al. [167] recorded high stability and better colloidal stability of iron oxide nanoparticles at high temperature (120 °C) via electrosteric stabilization using poly(AMPS-co-AA) copolymer. Zhao et al. [168] reported the good stability of PNF composed of starch and graphene nanoparticles. Similarly, Vasconcelos et al. [169] observed that ethylenediamine-modified graphene oxide nanoparticles exhibited good stability properties after aging for 90 days, with 146% higher viscosity over HPAM.

Furthermore, the presence of the nanoparticles in the polymer causes lower interfacial tension (IFT) of the oil–water interface, altering the wettability of the porous media and stabilizing emulsions [170,171]. Besides, the polymeric nanofluids show good emulsion stabilization properties. Corredor et al. [172] noted that polymeric nanofluid lowered the IFT of the oil–water interface by 66.7%. Sharma et al. [173] observed that the presence of nanoparticles in polyacrylamide causes the reduction in IFT at the oil–water interface. Bera et al. [174] studied the wettability behavior of nanoparticle-induced guar gum polymer and its suitability for oil recovery. The presence of the nanoparticle causes the reduction in contact angle from oil-wetting condition (115°) to water-wetting condition (72°). Additionally, Gbadamosi et al. [175] indicated that the use of aluminum nanoparticles with HPAM causes wettability alteration of the porous media. Saha et al. [176] investigated the emulsion properties of xanthan gum and silica nanoparticles. The polymeric nanofluid demonstrated excellent emulsion stabilization properties and was stable for a longer period. Pal et al. [177] investigated an emulsion stabilized by HPAM and SiO_2_ nanoparticles in the presence of a Gemini surfactant. The emulsion exhibited a more effective packing arrangement and good stability. Kumar et al. [178] concluded that emulsions stabilized with carboxymethylcellulose and SiO_2_ nanoparticles were stable over a wide range of temperature and lowered the IFT at oil–water interface.

The lowering of the IFT at the oil–water interface, alteration of wettability of the porous media, and improved rheological properties of polymeric nanofluids enhanced their efficiency in oil recovery. Oil displacement tests of several studies indicated incremental oil recovery of polymeric nanofluids when used as injectant in simulated sandstone and carbonate porous media. Keykhosravi et al. [179] reported the incremental oil recovery of anatase titanium oxide (TiO_2_)-induced xanthan gum solution in carbonate porous media. The polymeric nanofluid yielded an additional 25% OOIP. Moreover, Khalilinezhad et al. [180] reported an incremental oil recovery of polymer nanohybrid in a low-permeability carbonate oil reservoir. Bera et al. [174] observed an additional 17% OOIP with the synergic application of nanoparticles and guar gum over the conventional polymer flooding. Agi et al. [155] examined the oil recovery of PNF extracted from Cissus populnea in high-temperature high-pressure conditions and recorded 26% incremental oil recovery. Gbadamosi et al. [181] reported incremental oil recovery for xanthan gum containing SiO_2_, TiO_2_ and Al_2_O_3_ nanoparticles. Details from oil recovery studies with the use of polymeric nanofluid are presented in Table 6.

## 6. Economic Perspectives of Polymer Application for Chemical EOR

The economic implication of polymer injection for chemical EOR is profitable both in terms of oil recovery and reduction in water cut. By causing incremental oil recovery, the application of polymer floods becomes profitable on an economic scale. Meanwhile, a reduction in water cut by polymer flood implies a lower amount of money is expended on the treatment of produced water from the oil recovery process. Demin et al. [203] noted that the cost of waterflooding projects can be higher than that of polymer flooding projects based on actual field data. They surveyed field data of Daqing oilfield and noted that the total cost of injecting polymer is about 9 USD/bbl, which was equivalent to the cost of waterflooding in the same field. Furthermore, the water cut for waterflooding process for the field is about 90–95%, while the injection of polymers reduced the water cut to about 70%, and oil recovery increased to 4 times higher than that of waterflooding. With the current high oil price, the application of polymer flooding has become profitable. Currently, China tops the chart for the largest application of polymers for chemical EOR with more than 3000 wells and cumulative oil production of more than 300 million barrels [204]. The USA and Canada also have several wells (especially heavy oil reservoirs) implementing polymer application for chemical EOR.

## 7. Conclusions

Herein, the application of polymers for chemical enhanced oil recovery was appraised. The polymer types and mechanisms were discussed in detail. Additionally, the binary combination of polymers with other chemical additives for EOR was elucidated. Moreover, a synopsis of recent studies on polymer flooding applications was examined. Overall, HPAM remains the most coveted polymer despite the encouraging properties of other polymers. The application of polymers for chemical EOR requires careful screening of reservoir rock and fluid properties. The binary combination of polymers with other additives yielded positive results, and the deduced EOR types are beneficial for incremental oil recovery. The applications of polymers for EOR are mostly studied in sandstone. More research on tuning polymers for application in carbonate reservoirs is required. Scaling problems remain a major issue in the application of ASP which needs to be solved. There remains no consensus on the optimum injection slug for the SP flooding process. Moreover, few studies on rock–fluid interactions for polymeric surfactants exist in the literature, and the equilibrium phase behavior of polymeric nanofluids remains elusive. Further studies should consider studying the best injection slug for SP flooding; rock–fluid interactions of polymeric surfactants, especially in carbonate cores; and accurate modeling of polymeric nanofluid applications for EOR.

## Figures and Tables

**Table 1:** Polymer flooding screening criteria.

Reservoir Depth, ft	<9000	700–9460	NC	<5250
Porosity, %	NA	NA	NA	≥21
Permeability, mD	>10	1.8–5500	50	>1000
Oil viscosity, cP	10–100	0.4–4000	<150	<5400
Oil gravity, °API	>15	13–42.5	NC	>11
Oil saturation, %	>50	34–82	NA	>50
Temperature, °F	<200	<237	<200	<149
Salinity, ppm	NA	NA	<50,000	<46,000
Reference	[62]	[63]	[64]	[4]

**Table 2:** Merits and demerits of EOR polymers [65].

Polymer Type	Advantages	Disadvantages
HPAM	▪ Excellent solubility in water ▪ Tolerate mechanical shear	▪ Susceptible to temperature ▪ Precipitates in hard brines
HAPAM	▪ Excellent thickening capability ▪ Low retention in porous media	▪ Concentration regime dictates the polymer property
Xanthan gum	▪ Good thermal resistance ▪ Moderate shear stability ▪ Salinity and hardness resistance	▪ Highly susceptible to biodegradation ▪ High risk of plugging
Welan gum	▪ Exhibits long-term stability ▪ Good viscoelastic property	▪ Susceptible to inorganic cations present in reservoir brines
Guar gum	▪ Environmentally friendly polymer ▪ Shows excellent compatibility with salts ▪ Possesses good hydration properties	▪ Susceptible to temperature
Cellulose	▪ Possesses good resistance to mechanical shearing ▪ Shows good resistance to temperature	▪ Exhibits heterogeneous swelling ▪ Insoluble in water
Carboxymethylcellulose	▪ Environmentally friendly biopolymer ▪ Moderately soluble in water	▪ Suffers thermal degradation ▪ Prone to oxidative decomposition
Hydroxyethylcellulose	▪ High water solubility ▪ Good viscosifying effect ▪ Resistant to mechanical shearing and temperature	▪ High risk of biodegradation
Schizophyllan	▪ Excellent resistance to salinity and temperature ▪ Good thickening efficiency	▪ Highly susceptible to biodegradation
Scleroglucan	▪ High viscosifying property ▪ Resistant to thermal and shear effects	▪ Possesses poor filtering property in rock pores ▪ Susceptible to oxidation and biodegradation

**Table 3:** Summary of a few experimental studies on polymer flooding.

Polymer Type and Conc.	Experimental Condition(s)	Core Type	Rock Condition	Remarks	Ref.
HPAM HAPAM 4000 ppm	Brine salinity = 92,000 ppm, T = 82 °C, *μ_o_*= 1.6 mPa.s (@ 60 °C), Flow rate = 0.1 cc/min	Sandpack	*ϕ* = 24–27%, *k* = 2549 mD	The associative polymer recorded 6.52% incremental oil recovery over waterflooding as compared to 1.67% recorded by HPAM flooding. Hence, the associative polymer was recommended for pilot-scale test of South Turgay Basin.	[66]
HEC HAHEC 6000 ppm	*μ_o_* = 72 mPa.s (@ 50 °C), salinity = 15,296 mg/L, flow rate = 0.5 mL/min, temperature = 60 °C	Sandpack	*ϕ* = 32%	HAHEC displayed better viscosifying properties compared to HEC. Moreover, HAHEC lowered the IFT at the oil–water interface and caused emulsification of crude oil, which led to better oil recovery after waterflooding process.	[67]
HECT ragacanth gum HPAM	TDS = 5.567 g/L, oil viscosity = 0.31–0.48 (@ 45 °C)	Sandpack	*ϕ* = 35–36%	Incremental oil recovery of 7.38%, 6.71%, and 5.83% was recorded for HEC, tragacanth gum, and HPAM, respectively.	[68]
TVP PAM	TDS = 101,000 mg/L, Flow rate = 2 mL/min, temperature = 45 and 85 °C	Sandstone	*ϕ* = 20%, *k* = 200 mD	As compared to PAM which showed a monotonic decrease in viscosity, the thermoviscosifying polymer exhibited better thermothickening ability and salt tolerance. Oil displacement tests showed that TVP recorded higher oil recovery of 16.4% and 15.5% at 45 and 85 °C, respectively. PAM recorded 12.0% and 9.2% under the same conditions.	[69]
Guar gum	Temperature = 28 °C, oil viscosity = 24.8° API	Sandstone	*ϕ* = 15–39%, *k* = 206–248 mD	As compared to waterflooding, the use of guar gum resulted in an additional 20–26% incremental oil recovery.	[70]
Starch		Sandstone	*ϕ* = *~*23–27%, *k* = *~*291–293 mD	The application of starch biopolymers derived from waste material yielded 52–74% recovery from the sandstone cores.	[71]
Xanthan	Brine = 3.0 wt.%	Glassbead pack	*ϕ* = 36.9%, *k* = 3.79 darcys	The polymer exhibited good stability in high-salinity brine. Moreover, 3 wt.% concentration of the polymer yielded 30% incremental oil recovery over waterflooding.	[72]
HPAM	Brine = 3.0 wt.%, temperature = 25 °C oil viscosity = 450 cP, flow rate = 4 mL/min	Glassbead pack	*ϕ* = 37%, *k* = 3.4 darcys	Oil displacement results revealed that the application of HPAM resulted in approximately 22% incremental oil recovery over waterflooding process.	[51]
Welan gum Xanthan gum	Temperature = 50 °C, flow rate = 0.5 mL/min, salinity = 9374 mg/L, oil viscosity = 458 cP (@ 50 °C)	Sandpack	*ϕ* = 38%, *k* = 0.18–1.51 μm^2^	At the same concentration, the elastic and viscous modulus of welan gum were higher than xanthan gum. Moreover, the core flooding results showed that welan gum recorded 7.3% and 25.4% additional oil recovery over xanthan gum and waterflooding, respectively.	[42]
Schizophyllan	Oil viscosity = 35 cP, salinity = 180 g/L, temperature = 55 °C)	Sandstone	*ϕ* = 24%, *k* = 1900 mD	The injection of schizophylan yielded good oil recovery and residual resistance factor.	[73]
Scleroglucan ATBS	TDS = 3800 mg/L, oil viscosity = 390 cP (@ 100 °C).	Sandstone	*ϕ* = 18.8–20.4%	As compared to the sulfonated polyacrylamide (2500 mg/L), scleroglucan (935 mg/L) recorded approximately 10% incremental oil recovery.	[74]

**Table 4:** Summary of recent experimental studies on surfactant–polymer flooding process.

Surfactant Type	Polymer Type	System Type	Rock Type	Exp Conditions	Findings	Ref.
Sodium dodecylbenzenesulfonate (SDBS)	Carboxymethyl cellulose	SP	Sandpack	Flowrate = 0.5 mL/min	The injection of SP slug resulted in 14–20% incremental oil recovery. The incremental oil recovery was attributed to factors such as emulsion generation, IFT reduction, and optimum viscosity of the SP slug.	[112]
SDBS	HPAM	SP	Sandstone	Flow rate = ~1 ft/day, brine = 40 g/L NaCl	The use of polymers in SP flooding reduced the PV of the injectant. Homogeneous formulation of SP flooding recovered 66% OOIP. Moreover, the use of a homogeneous SP system reduced the adsorption of the surfactant on rock pores.	[127]
Alkoxysulfate	HPAM	SP	Sandpack	Oil viscosity = 6.6 cP (@ 55 °C), formation water salinity = 107.83 g/L	A low concentration (500 ppm) of surfactant was found to enhance the oil recovery efficiency of the polymer flood by 13% OOIP. Moreover, the authors suggested that the optimal salinity of the surfactant show be greater than that of the injected water. Ultralow surfactant concentration was recommended to avoid issues associated with high surfactant concentration, such as persistent emulsions and aqueous solubility.	[128]
Anionic surfactant Nonionic surfactant	HPAM	SP	Sandpack	Oil viscosity = 1300 cP, Oil density = 970.1 kg/m^3^, flow rate = 0.001 mL/min, temperature = 70 °C	SP demonstrated good emulsion stability. The injection of 0.5 PV of SP flood resulted in 30.7–32.7% incremental oil recovery.	[107]
Soap-nut surfactant 8000–10,000 ppm	HPAM 1000 ppm	SP	Sandpack	Oil viscosity = 18.9 ° API, 2 wt.% brine solution	The IFT of the solution decreases with an increase in the surfactant concentration. Moreover, the presence of the surfactant altered the wettability of the sandstone rock surface from 83.5° to 20.8°. The adsorption of the natural surfactant on quartz surface was low due to electrostatic repulsion. SP flooding process recorded approximately 30% incremental oil recovery with different slug injections.	[129]
Polyether carboxylate anionic nonionic surfactant	HMPAM, HPAM	SP	Sandstone	Oil viscosity = 562.4 cP (@ 65 °C), oil density = 0.963 g/cm^3^, pressure = 10 MPa, temperature = 65 °C	The application of SP flooding yielded 15.54% incremental heavy oil recovery. The synergic combination of polymer flooding and SP flooding yielded 40.64% incremental oil recovery.	[130]
Soldium allyl-sulfonate, acrylic ester, allyl glycidyl ether	Acrylamide	Polymeric surfactant	Sandstone	Flow rate = 0.8 mL/min, temperature = 55 °C	As compared to polymer (HPAM) flooding that resulted in 11.5% incremental oil recovery after waterflooding process, the use of polymeric surfactant flooding achieved 17.5% incremental oil recovery.	[123]
Sodium methyl ester sulfonate	Acrylamide	Polymeric surfactant	Sandpack	Oil viscosity = 23.11 °*API*, 40 cP (@ 30 °C)	The polymeric surfactant reduced IFT at oil–water interface to 0.37 mN/m at the optimum salinity. Besides, the polymeric surfactant exhibited shear thinning behavior. Finally, 26% incremental oil recovery over conventional waterflooding was recorded during the flooding of sandpack.	[117]
Sodium methyl ester sulfonate	Acrylamide	Polymeric surfactant	Sandstone	Flow rate = 1.83 mL/s	The synthesized polymeric surfactant reduced the contact angle of oil-wet quartz surface to 25.47° after 10 min contact time. The IFT of the oil–water interface was also reduced to 2.3 mN/m. Finally, a total recovery of 77.98% was achieved by the injection of the polymeric surfactant.	[131]

**Table 5:** Summary of recent experimental studies on alkali–surfactant–polymer flooding.

Alkali Type	Surfactant Type	Polymer Type	Experimental Condition(s)	Rock Type	Finding	Ref.
Na_2_CO_3_	Alkylbenzene sulfonate, fatty alcohol propoxylated sulfate, cocamidopropyl hydroxysultaine	HPAM (MW = 20 × 10^6^)	Formation brine (7500 ppm TDS), injection brine (5300 ppm) oil viscosity = 60 cp (@ 62 °C)	N/A	Injection of 0.3 PV of ASP slug resulted in incremental oil recovery of 44.5% over waterflooding.	[135]
NaOH (2500 ppm)	Anionic surfactant from waste chicken fat (5500 ppm)	*Hollyhocks* (2000 ppm)	Oil viscosity = 41.34 cP (@ 15.56 °C), temperature = 80 °C, flow rate = 0.2 mL/min, salinity = 62,000 TDS	Sandstone	The novel polymer solution non-Newtonian behavior. Moreover, 27.9% incremental oil recovery was achieved with the use of ASP slug injection into sandstone.	[143]
Na_2_CO_3_	Carboxybetaine zwitterionic surfactant	HPAM	Oil viscosity = 30 °API	Sandpack	The surfactant altered the permeability of the oil-wet quartz sample. The experimental result from sandpack flooding indicates the ASP slug injection recovered 30.82% OOIP.	[146]
NaOH	Anionic surfactant from waste chicken fat (5500 ppm)	HPAM (1000 ppm)	Flow rate = 0.2 mL/min, temperature = 75 °C	Carbonate	The alkali–surfactant mixture reduced the IFT and altered the wettability of the carbonate from oil-wet to water-wetting condition. For the application of ASP in carbonate, 17.8% incremental oil recovery was recorded.	[147]
NaOH	SDBS	HPAM	–	Sandstone	Ultralow interfacial tension was generated using a very low concentration of alkali and surfactant while the injected polymer enhances the mobility control. An additional 20% OOIP over conventional waterflooding was found.	[148]
Ethoxylated diisopropylamine	Carboxylate and sulfonate surfactant	HPAM (3330S)	Salinity = 60,000 ppm, temperature = 100 °C	Carbonate	ASP yielded ultralow IFT, low surfactant retention, and high recovery in carbonate cores characterized by high permeability, nonfracture, and HTHS condition. Cumulative oil recovery using ASP slug ranges from 85.2 to 93.6%.	[149]
NaBO_2_ NH_4_OH	Isobutyl alcohol-3-ethoxylate, internal olefin sulfonate	HPAM 3630S, 3330S, AN 125	Formation brine = 147,507 ppm, hardness = 2144 ppm (*Ca^2+^, Mg^2+^*), injection brine = 1–3 wt.% NaCl	Carbonate and Sandstone	The use of sodium metaborate and ammonium hydroxide as alkalis in the ASP corefloods yielded low surfactant retention and high oil recoveries.	[150]
Na_2_CO_3_ NaOH	PS, IOS, IBA-EO, TSPC, EPS	HPAM (FP 3330S) 3500 ppm	Oil viscosity = 8 cP, NaCl = 22,390 ppm, Na_2_SO_4_ = 2464 ppm, CaCl_2_.2H_2_O = 983 ppm, and MgCl_2_.6H_2_O = 2340 ppm	Limestone cores	The study revealed that the pore throat radii of the rock must be bigger than the polymer hydrodynamic radius for successful polymer transport. Moreover, the secondary application of ASP yielded 77–87% cumulative OOIP in low-permeability rocks.	[137]
Triethylamine	Sodium ethyl ester sulfonate (SEES)	HPAM	Oil viscosity = 23.55 °API (30 °C), Brine = 1 wt.%	Sandpack	Alkali and surfactant played a crucial role in the IFT reduction to ultralow values. Besides, 34.79% incremental oil recovery was achieved with 0.2 wt.% HPAM and 0.8 wt.% SEES.	[151]
Ethanolamine	Sulfonate-based surfactants	HPAM (1000 ppm)	Salinity = 13,659.9 ppm (NaCl, MgCl_2_, and CaCl_2_)	Sandpack	The use of organic alkali resulted in ultralow IFT, stable oil-in-water emulsion, and enhanced oil displacement efficiency. Moreover, approximately 20% incremental oil recovery was recorded during sandpack flooding.	[138]
Monoethylamine NaOH		*Tragacanth* gum	Viscosity = 31.14 *° API*, formation water salinity = 74,000 TDS, temperature = 75 °C	Carbonates	The synthesized polymeric surfactant increased the viscosity of water and reduced the mobility ratio of the injectant. Moreover, IFT was reduced to 2.329 mN/m at the optimum salinity conditions. For the ASP flooding, 21.4% incremental oil recovery was recorded.	[122]
NaOH Na_2_CO_3_	Alkylbenzene sulfonate	HPAM (MW = 25 × 10^6^)	Oil viscosity = 9.8 cP (@ 45 °C)	Sandstone	The study showed that the viscosity, IFT, and hydrodynamic diameter of ASP containing weak alkali surpassed those of strong alkali at the same concentration. ASP containing weak alkali had 22% incremental oil recovery.	[152]

**Table 6:** Oil recovery from displacement tests of polymeric nanofluids.

NP Type	Polymer/Copolymer Type	PNF Conc.	Brine/Conc.	Temp	Porous Medium Type	Incremental Oil Recovery (%)	Reference
SiO_2_, Al_2_O_3_	HPAM	100–2500 ppm	0.6 wt% KCl	–	Sandpack	5.0–9.0	[182]
SiO_2_	PEOMA	10,000 ppm	1.0 wt.% NaCl	30 °C	Berea sandstone	19.5	[183]
APTES-SiO_2_ OTES-SiO_2_	HPAM	625 ppm NP 2500 ppm HPAM	2000–10,000 ppm	90 °C	Sandstone core	4.6–12.3	[184]
SiO_2_	PAMAM	1500 ppm	10 wt.% NaCl, 0.15 wt.% MgCl_2_ 0.10 wt.% CaCl_2_	90 °C	Berea Sandstone	16.3	[185]
Graphene	Gum arabic	50 ppm	3.0 wt.% NaCl	90 °C	Berea sandstone	17.12	[186]
SiO_2_	Prop-2-enamide/AM	8000 ppm	–	80 °C	Quartz sand	21.0	[187]
GO	HPAM	0.2 wt.% NP 0.05 wt.% HPAM		25 °C	Sandpack	7.8	[188]
SiO_2_	AMPS	50,000 ppm	–	80 °C	Quartz sand	23.22	[189]
Al_2_O_3_	Potato starch Gum arabic	1.3 wt% 3–5 wt.%	3.0 wt.% NaCl	25 °C	Sandstone	5.16–7.18	[190]
SiO_2_	PEG	10,000 ppm	–	80 °C	Glass micromodel	20.0	[191]
SiO_2_ Al_2_O_3_ TiO_2_	Xanthan gum	5000 ppm	3.0 wt.% NaCl	80 °C	Sandstone	7.2–11.2	[181]
SiO_2_	MeDiC_8_AM	1500 ppm	12 wt.% (NaCl & CaCl_2_)	82.3 °C	Sandstone	20.0	[192]
SiO_2_	AMC_12_S	1100 ppm	18 wt.%	110 °C	Sandstone	24.0	[193]
ZnO/SiO_2_	Xanthan	2000 ppm	1660 ppm	75 °C	Carbonate	19.28	[194]
SiO_2_	AA/AM	2000 ppm	2 wt.% NaCl, 0.18 wt.% CaCl_2_	65°C	Sandstone	20.1	[195]
SiO_2_	PA–S	3000 ppm	5 wt.% NaCl, 2 wt.% CaCl_2_	25 °C	–	12.77	[196]
SiO_2_	AM/AA	1500 ppm	–	–	–	18.84	[197]
SiO_2_	HPAM	1000 ppm	2.4 wt.% (NaCl, CaCl_2_, MgCl_2_)	25 °C	Glass micromodel	10.0	[198]
SiO_2_	HPAM	800 ppm	3 wt.% NaCl	–	Glass micromodel	10.0	[199]
TiO_2_	HPAM	–	2 wt.% (NaCl, CaCl_2_, MgCl_2_.6H_2_O, Na_2_HCO_3_)	–	Sandstone	4.0 *	[200]
MMT Clay	HPAM	1000 ppm	10 wt.% (NaCl, CaCl_2_, MgCl_2_)	90 °C	Quartz sand	33.0	[157]
SiO_2_	Guar gum	0.2 wt.% NP 4.0 wt.% guar gum	–	50 °C	Sandstone	12.95	[174]
SiO_2_	Xanthan	0.3 wt.% NP 5000 ppm XG	4445 ppm	30 °C	Sandstone	20.82	[176]
SiO_2_ Clay	HPAM	1500 ppm	2.0 wt.% (NaCl, CaCl_2_, MgCl_2_.6H_2_O)	–	Sandstone	13.0	[201]
SiO_2_	HPAM	600 ppm	6.0 wt.% (NaCl, CaCl_2_, MgCl_2_.6H_2_O Na_2_SO_4_ Na_2_HCO_3_)	80 °C	Quartz sand	10.54	[202]

* Heavy oil.

## Nomenclature

AA	Acrylic acid
AM	Acrylamide
AMC_12_S	2-Acrylamido-dodecyl sulfonate
APTES	(3-Aminopropyl)triethoxysilane
ATBS	Sodium acrylamido terbutyl sulfonate
AN125	Hydrolyzed acrylamido propyl sulfonated acid
HEC	Hydroxyethyl cellulose
HAHEC	Hydrophobically associating hydroxyethyl cellulose
HMPAM	Hydrophobically modified polyacrylamide
MeDiC_8_AM	2-Methyl-*N*,*N*-dioctyl-acrylamide
NaBO_2_	Sodium borate
Na_2_CO_3_	Sodium carbonate
NH_4_OH	Ammonium hydroxide
OTES	Octyltriethoxysilane
PEG	Polyethylene glycol
TVP	Thermoviscosifying polymer
